# Dual Exposure to E-Cigarette Vapour and Cigarette Smoke Results in Poorer Airway Cell, Monocyte, and Macrophage Function Than Single Exposure

**DOI:** 10.3390/ijms25116071

**Published:** 2024-05-31

**Authors:** Rhys Hamon, Leigh Thredgold, Asiri Wijenayaka, Nicole Anne Bastian, Miranda P. Ween

**Affiliations:** 1 Centre for Cancer Biology, SA Pathology and the University of South Australia, Adelaide, SA 5000, Australia; 2 Department of Thoracic Medicine, Royal Adelaide Hospital, Adelaide, SA 5000, Australia; 3 School of Medicine, University of Adelaide, Adelaide, SA 5005, Australia; 4 Department of Occupational and Environmental Health, School of Public Health, University of Adelaide, Adelaide, SA 5005, Australia

**Keywords:** e-cigarettes, dual user, cigarettes, macrophages, monocytes, toxicity, migration, phagocytosis, respiratory

## Abstract

E-cigarette users predominantly also continue to smoke cigarettes. These Dual Users either consume e-cigarettes in locations where smoking is not allowed, but vaping is, or to reduce their consumption of cigarettes, believing it will lead to harm reduction. Whilst it is known that e-cigarette vapour is chemically less complex than cigarette smoke, it has a distinct chemical profile, and very little is known about the health impacts of exposure to both chemical profiles vs. either alone. We simultaneously exposed cells in vitro to non-toxic levels of e-cigarette vapour extract (EVE) and cigarette smoke extract (CSE) to determine their effects on 16HBE14o- airway epithelial cell metabolism and inflammatory response, as well as immune cell (THP-1 cells and monocyte-derived macrophages (MDM) from healthy volunteers) migration, phagocytosis, and inflammatory response. We observed increased toxicity, reduced metabolism (a marker of proliferation) in airway epithelial cells, and reduced monocyte migration, macrophage phagocytosis, and altered chemokine production after exposure to either CSE or EVE. These cellular responses were greater after dual exposure to CSE and EVE. The airway epithelial cells from smokers showed reduced metabolism after EVE (the Switcher model) and dual CSE and EVE exposure. When EVE and CSE were allowed to interact, the chemicals were found to be altered, and new chemicals were also found compared to the CSE and EVE profiles. Dual exposure to e-cigarette vapour and cigarette smoke led to worse functional outcomes in cells compared to either single exposure alone, adding to limited data that dual use may be more dangerous than smoking only.

## 1. Introduction

E-cigarettes have been shown to cause a widening range of effects, beyond toxicity, in cells, animal models, and humans (reviewed in Bozier et al. [1]). The effects observed in the lung include enlarged human lung fibroblasts with induced spindle formation and vacuolization [2] and the loss of lung endothelial barrier function [3]. Bozier et al., the National Academies of Sciences, Engineering, and Medicine review, and Banks et al. have provided excellent systematic reviews on the known health effects of e-cigarettes using in vitro, ex vivo, and in vivo studies [1,4,5]. Data from previous studies, including our own, are forming a clear picture that even nicotine-free e-liquids may not be harmless, likely because of the flavourants present [1,4,6,7,8].

One of the largest groups of e-cigarette users are those who smoke tobacco cigarettes and vape e-cigarettes, called “Dual Users” [9,10]. Some of the reasons for dual use are reduced cost, the ability to use e-cigarettes in places where smoking tobacco cigarettes is not allowed, greater enjoyment of e-cigarette use, and perception of harm reduction to themselves and others [11]. However, this latter notion is based on a simple assumption that tobacco cigarettes (a) contain a larger number of toxic chemicals than e-cigarettes and (b) involve inhaling combustion products, whilst e-cigarettes do not. The belief is that therefore, dual use exposes the user to fewer toxic chemicals overall and thus must, logically, pose less harm. However, this line of thought ignores the fact that the chemical profiles of vapour from e-cigarettes are very different from those of smoke from tobacco cigarettes and could act on different molecular pathways to induce negative health effects. Thus, dual use could potentially lead to worse health outcomes compared to smoking or vaping alone. Early data in the literature provide evidence that this could indeed be the case. Dual use leads to exposure to a higher diversity of toxicants and an increased risk of heart attack, stroke, asthma, and cancer compared to exclusive smokers or vapers [12,13,14,15]. Further, both cigarette smoke and e-cigarette vapour are known to contain highly reactive compounds [16,17,18,19,20], which, upon combination, could easily form unique chemical adducts that dissolve into the fluid of the lung luminal space, leading to the activation of entirely new molecular pathways.

This study presents a thorough in vitro analysis with ex vivo validation that compares the impacts of dual exposure vs. single exposure on airway cells, including toxicity, metabolism, cytokine production, and immune cell function. We additionally compared the chemical profiles of dual versus single e-cigarette and cigarette smoke extracts to identify changes resulting from the interaction of tobacco smoke with e-cigarette vapour.

## 2. Results

### 2.1. Dual Exposure of Bronchial Epithelial Cells Results in Increased Toxicity and Impaired Proliferative Capacity

**Figure 1:**
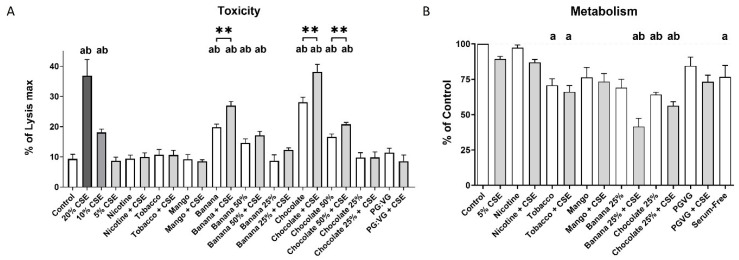
Dual exposure of 16HBE cells. The 16HBE cells were exposed to EVE, CSE, or EVE + CSE for 24 h. (**A**) LDH release from 16HBE cells. (**B**) Conversion of MTT to formazan by 16HBE cells, relative to control. Unless indicated otherwise, 100% EVE and 5% CSE were used. N = 6. Mean ± SEM. a: *p* < 0.05 compared to control; b: *p* < 0.05 compared to 5% CSE, ** *p* < 0.01 between pairs.

We have previously reported that 10% CSE, 100% banana EVE, and 100% chocolate EVE were sufficient to cause increased 16HBE cell death when full monolayers were exposed, and that the LDH assay proved to be very high sensitivity toxicity assay [8]. We first performed experiments to identify non-toxic single and dual exposure concentration in the 16HBE cells so that cell metabolism, as measured by MTT conversion as a marker of proliferative capacity, would not be confounded by the dying cells. We found that reductions to 5% CSE, 25% banana EVE, and 25% chocolate EVE were required to prevent significant toxicity, whether alone or as a dual exposure, as measured by LDH release. Increased toxicity was observed when the cells were dually exposed to 100% and 50% banana or chocolate EVE with 5% CSE vs. 5% CSE alone. Additionally, increased toxicity was observed upon dual exposure to 100% banana EVE with 5% CSE or 100% and 50% chocolate EVE with 5% CSE vs. the respective EVE alone (Figure 1A). Exposure to 100% nicotine, mango, and PGVG EVE did not induce an increased release of LDH alone or in dual combination with 5% CSE.

Using the cellular metabolism of MTT to formazan as a proxy for proliferative capacity, a key attribute for wound healing, the 16HBE cells displayed reduced proliferative capacity after exposure to tobacco EVE, dual tobacco, dual banana, chocolate, and dual chocolate, relative to control. Interestingly, individual exposure to CSE and banana was not enough to significantly reduce the proliferative capacity of the 16HBE cells compared to control; however, when combined, a significant reduction was observed compared to both the control and CSE treatments. Dual banana and dual chocolate exposures also displayed proliferative capacity significantly below to that of CSE exposure alone.

### 2.2. Models of Dual Use and Switching Using Smoker NHBE Show Reduction in Proliferative Capacity

As NHBE cells from smokers (SHNBE) were commercially available and able to be passaged to expand to the numbers needed for this work, they were used to model the impact of both “Dual User” and “Switcher” usage habits, whereby cigarette smokers either transition to dual use with e-cigarettes or switch to exclusive e-cigarette use.

**Figure 2:**
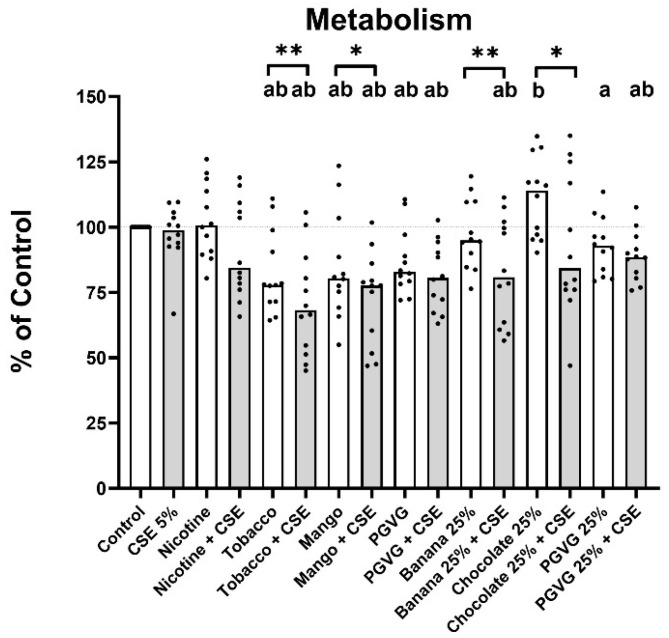
Proliferative capacity of Smoker bronchial epithelial cells in a Switcher and Dual-User model. Conversion of MTT to formazan was measured in SNHSBE cells exposed to EVE (Switcher), EVE + CSE (Dual User), or controls for 24 h. Unless indicated otherwise, 100% EVE and 5% CSE were used. Data are plotted as the median plus individual data points. a: *p* < 0.05 compared to control. b: *p* < 0.05 compared to 5% CSE, * *p* < 0.05 between pairs ** *p* < 0.01 between pairs.

In the Dual-User model, exposure to 5% CSE showed no significant decrease in proliferative capacity compared to control, likely due to the exposure of the cells to cigarette smoke prior to collection. Exposure to dual tobacco, dual mango, dual banana, and dual PG:VG significantly decreased the proliferative capacity of SNHBE cells below that of both CSE-only and their matching EVE-only exposures. Dual chocolate exposure had a significantly decreased proliferative capacity compared to chocolate EVE-only but was not significantly less than the CSE-only exposure (Figure 2).

In the Switcher model, exposure to tobacco EVE or mango EVE significantly reduced proliferative capacity below control. However, this is likely due to the fact that PG:VG alone caused a significant reduction in proliferative capacity compared to control, which persisted even at a 25% PG:VG EVE concentration. Interestingly, exposure to nicotine EVE, banana EVE, and chocolate EVE had no significant reduction compared to control, despite the presence of PG:VG.

Thus, comparisons were further assessed against the matching PG:VG EVE exposure or dual PG:VE + CSE exposure (Table 1). In the Dual-User model, the proliferative potential was significantly decreased upon exposure to dual nicotine, dual tobacco or dual mango compared to dual PGVG, whilst in the Switcher model, the proliferative potential was significantly higher in nicotine, banana, and chocolate EVE-exposed cells when compared against exposure to the e-liquid base: PGVG EVE.

### 2.3. Dual Exposure Induces Decreased Migration and Spontaneous Differentiation to Macrophages in THP-1 Monocytes

**Figure 3:**
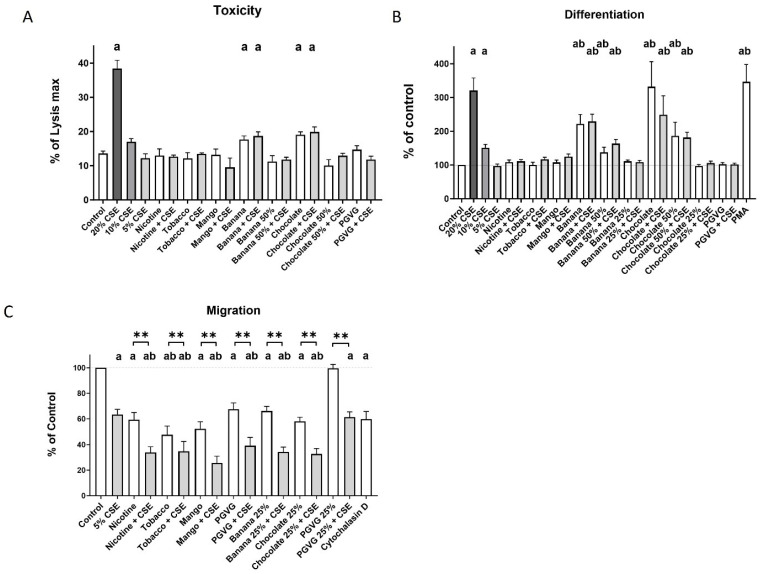
Dual exposure of THP-1 monocytes. THP-1 monocytes were seeded in CSE, EVE, or EVE + CSE and (**A**) LDH release, (**B**) differentiation, and (**C**) migration was measured. Unless indicated otherwise, 100% EVE and 5% CSE were used. N = 6. Mean ± SEM. a: *p* < 0.05 compared to control. b: *p* < 0.05 compared to 5% CSE, ** *p* < 0.01 between pairs. PMA was used as a positive control for differentiation, and Cytochalasin D was used as a negative control for migration.

Experiments to identify non-toxic single and dual exposure conditions for THP-1 monocytes were conducted prior to the migration assay to prevent any confounding by the presence of dead/dying cells (Figure 3A). There was no difference in toxicity noted between single and dual exposures.

We also assessed whether the exposures induced spontaneous differentiation that resulted in adherence, so that the migration assays would not be confounded by differentiating cells. Exposure to 20% CSE, 100% banana EVE, dual 100% banana, 100% chocolate EVE, and dual 100% chocolate increased THP-1 monocyte toxicity, as measured by LDH release, and also induced differentiation/adherence. Exposure to 10% CSE, 50% banana, dual 50% banana, 50% chocolate, and dual 50% chocolate was not toxic but did induce differentiation compared to control (Figure 3B). Thus, 5% CSE, 25% banana, and 25% chocolate were used for the migration assays.

Exposure to 5% CSE, nicotine EVE, and all EVE flavours alone or dual with CSE reduced THP-1 cell migration compared to control (Figure 3C). Dual nicotine and all dual flavours also reduced THP-1 cell migration below that of 5% CSE exposure. Significant reductions in migration were noted between every dual exposure and its single exposure counterpart. Interestingly, 100% PGVG also reduced migration, but 25% PGVG did not. Thus, further statistical analysis was performed against the matching PG:VG controls (Table 2). When compared against the corresponding PGVG exposure, THP-1 migration was significantly decreased by exposure to nicotine, tobacco, mango, banana, dual banana, chocolate, and dual chocolate migration.

### 2.4. Dual Exposure Decreases Normal Healthy Monocyte Migration and Further Exacerbates Decreased Phagocytic Capacity

**Figure 4:**
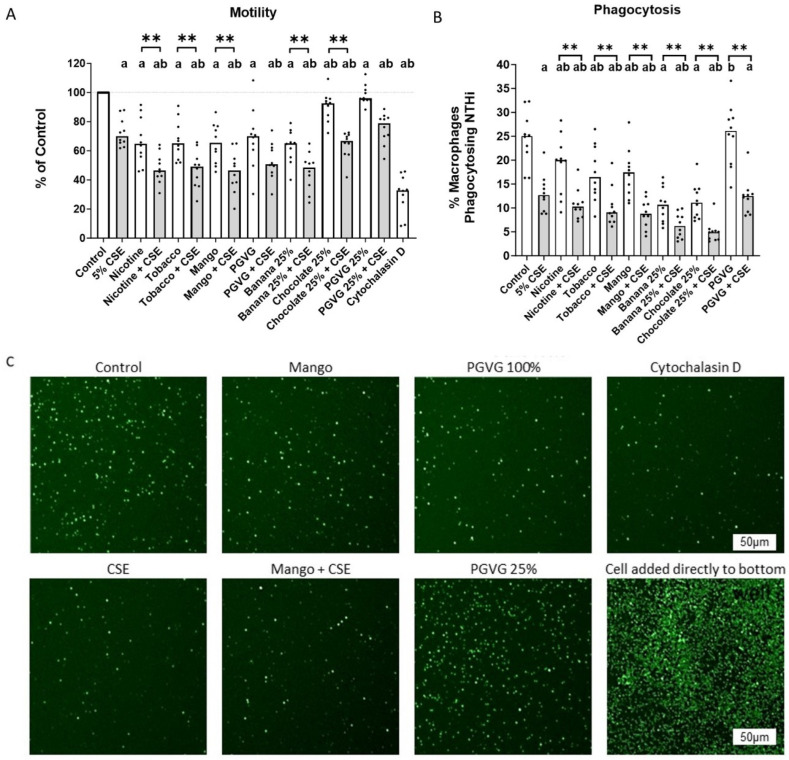
Effects of dual exposure on monocyte migration and MDM phagocytosis. (**A**) Healthy non-smoker donor monocytes were exposed to CSE, EVE, or EVE + CSE, and monocyte migration was assessed. (**B**) Monocyte-differentiated macrophages from healthy non-smokers were exposed to CSE, EVE, or EVE + CSE, and their ability to phagocytose NTHi bacteria was assessed. (**C**) Examples of healthy monocytes migrated to lower well via microscopy. Unless indicated otherwise, 100% EVE and 5% CSE were used. The data show the median value plus individual data points. a *p* < 0.05 compared to control. b *p* < 0.05 compared to 5% CSE, ** *p* < 0.01 between pairs.

Exposure to CSE, nicotine, and all individual EVE flavours alone or dually with CSE reduced donor monocyte migration compared to control. Dual nicotine and all dual exposure treatments also reduced migration compared with CSE alone. When comparing single vs. dual exposure matching pairs, Nicotine EVE and all flavours EVE showed a significant decrease with dual exposure vs. single EVE exposure (Figure 4A). As observed in THP-1 monocytes, 100% PGVG also reduced migration, while 25% PGVG did not. Therefore, further statistical analysis against the corresponding PG:VG controls was performed (Table 3). When compared with their matching PGVG control; dual nicotine, dual tobacco, dual mango, banana, dual banana, chocolate, and dual chocolate showed further decreased migration. This was also visualised via microscopy (some representative images are shown in Figure 4C). Cytochalasin D was used as a negative control for migration.

We have previously reported that CSE nicotine and flavoured EVE could reduce the alveolar macrophage phagocytosis of bacteria [7]. Healthy donor monocyte-derived alveolar-like macrophages were used as a model for alveolar macrophages due to the numbers required for dual exposure analysis. CSE, nicotine, dual nicotine, and all single and dual flavours reduced phagocytosis compared to control, as expected. PGVG did not affect phagocytosis. Dual nicotine and all dual-flavoured EVEs reduced phagocytosis compared to CSE alone. When comparing single vs. dual exposures, nicotine and all flavoured EVEs showed a significant reduction in phagocytosis in the dual exposure vs. the single exposure (Figure 4B).

### 2.5. Dual Exposure Resulted in Greater Disruption of Macrophage Cytokine Production Than Single Exposure

**Figure 5:**
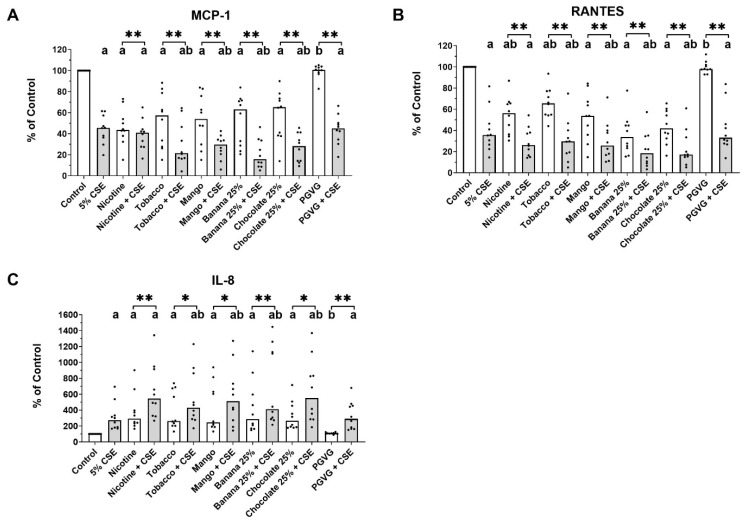
Cytokine secretion after dual exposure of healthy non-smoker MDMs. Monocyte-differentiated macrophages from healthy non-smokers were exposed to CSE, EVE, or EVE + CSE, and secreted (**A**) MCP-1, (**B**) RANTES, and (**C**) IL-8 were measured. Unless indicated otherwise, 100% EVE and 5% CSE were used. The data show the median plus individual data points. a: *p* < 0.05 compared to control. b: *p* < 0.05 compared to 5% CSE, * *p* < 0.05 between pairs. ** *p* < 0.01 between pairs.

Exposure to CSE, nicotine EVE, and all flavoured EVEs reduced the secretion of MCP-1 and RANTES, while increasing secretion of IL-8 from MDMs compared to the control exposure. All flavours in dual EVE exposure further enhanced this change compared to CSE alone, whilst dual nicotine-only enhanced this change compared to CSE for RANTEs and IL-8, but not for MCP-1. PGVG had no effect on MPC-1, RANTES, or IL-8 secretion. Dual nicotine and all EVE flavours in dual EVE exposure showed a further significant deviation from their corresponding single EVE exposure for all three cytokines (Figure 5).

### 2.6. Interaction between Cigarette Smoke and E-Cigarette Vapour in the Soluble Phase Results in Changes in VOCs

One possible reason for the increased effects of dual exposures vs. single exposure may be due to the interaction of cigarette smoke with e-cigarette vapour. We modified our existing extract production method to recapitulate, in a soluble phase, what would happen in the lung lining fluid of a Dual User. The preparation of 100% CSE was first made, and then, immediately after, either 100% chocolate or 100% PGVG e-cigarette vapour was bubbled through the CSE. The reverse procedure was also performed. Samples were either frozen immediately or incubated at 37 °C with 5% CO_2_ for 24 h to allow for longer interaction (aged). Dual extracts were then VOC profiled and compared against 100% CSE, 100% chocolate EVE, and 100% PGVG EVE.

Firstly, we observed that the PG:VG sample, which was made from PG and VG stocks purchased from a vape store, had chemicals present that were not present in the chocolate sample, therefore it was not suitable for comparing with the chocolate single-extract samples (Table S1).

In the extracts frozen immediately after collection, we observed aniline and 2,2′-Bipyrazine in the CSE made into the chocolate EVE, and 1-ethoxy-2-methylbenzene, 3-pyridinamine, phosphoryl fluoride, and N-methyl-benzeneacetamide in the chocolate EVE made into CSE, which were not present in CS or chocolate single extracts. We further observed 2-Pentanone, acetic acid ethenyl ester, methylpyrazine, 2-ethenyl-pyridine, 3-methyl-2-cyclopenten-1-one, 1-methoxy-4-methyl-benzene, 2-oxo-3-cyclopentene-1-acetaldehyde, and 3-methylpyridine in CS or chocolate single extracts that were not detected in the CSE made into the chocolate EVE. Additionally, 2-Pentanone, acetic acid ethenyl ester, methylpyrazine, 2-ethenyl-pyridine, 3-methyl-2-cyclopenten-1-one, 1-methoxy-4-methyl-benzene, cyclopentanone, 2,2-dimethyl-propanal, allyl pentyl ester oxalic acid, 4-ethylphenol, and 2,5,6,7-tetrahydro-3H-Cyclopenta[c]pyridazin-3-one/3-methylene-2-oxo-cyclohexanecarboxylic acid, methyl ester were found in CS or chocolate single extracts that were not detected in the chocolate EVE made into the CSE (Table 4). This demonstrates the rapid generation of new VOCs and the likely alteration/degradation of VOCs due to the interaction of the chemicals in CSE with chocolate EVE.

In the extracts allowed to further interact at 37 °C with 5% CO_2_ for 24 h after collection, we observed pyrrole, 3-methyl-2-cyclopenten-1-one, and cyclopentanone in the CSE made into the chocolate EVE and pyrrole and 3-methyl-2-cyclopenten-1-one in the chocolate EVE made into CSE, which were not present in CS or chocolate single extracts. We further observed methylpyrazine, methyl alcohol, methylglyoxal, and 3-pyridinol, in CS or chocolate single extracts that were not detected in the CSE made into the chocolate EVE. In addition, methylpyrazine, methyl alcohol, methylglyoxal, and 3,4-dimethyl-2-cyclopenten-1-one/2,3-dimethyl-2-cyclopenten-1-one were found in CS or chocolate single extracts that were not detected in chocolate EVE made into the CSE (Table 5). This demonstrated that the interaction of the chemicals in CSE and chocolate EVE over time at physiological temperatures results in the generation of new VOCs and the alteration of VOCs, which was mostly different from the changes immediately after the interaction of the two extracts.

The changes between PGVG EVE and interacting CSE and PGVG EVE are documented in Supplemental Tables S1 and S2.

When we compared freshly collected extracts vs. aged extracts, we also observed the detection of new VOCs, as well the disappearance of many others, likely due to alterations such as thermal decomposition or degradation (Tables S3–S9).

## 3. Discussion

Whilst e-cigarettes have only been mainstream for about 10–15 years, there has been a recent surge in promoting them as a form of harm reduction, resulting in a large number of smokers who “Switch” to e-cigarette use, 30–40% of whom continue to use e-cigarettes long term ≥ 1 yrs [21,22], and those who “Dual use”, both smoking and vaping [9,10].

Whilst there is no dispute about the harms of smoking cigarettes, and data continue to mount against vaping being harm-free [23], including a growing body of evidence suggesting that the harms of vaping extend beyond the lungs [24,25,26,27,28], we still understand very little about dual use. Dual use exposes users to two different sets of chemicals, which potentially activate different molecular pathways, leading to increased harm, a very different scenario to the expected harm reduction many users believe they are achieving via dual use.

We and others have previously shown that flavouring chemicals, in particular, contribute to e-cigarettes’ negative health impact [6,7,8,29]. Thus, in this study, we investigated the impacts of dual exposure to four different flavoured e-liquids, with and without added cigarette smoke, including: two with previously observed high cellular impact and two observed to have lower cellular impacts [7,8].

We have previously shown that the detection of LDH release was a more sensitive indicator of bronchial epithelial cell toxicity than annexin V or nucleic acid staining [6,7], so this parameter was utilised to determine the non-toxic doses of EVE and CSE. Toxicity to bronchial epithelial cells was assessed by the loss of 16HBE membrane integrity and subsequent LDH release, which showed that dual exposure to two out of four flavoured EVEs and CSE was worse than exposure to CSE or to flavoured EVE alone. Whilst toxicity due to CSE or EVE was expected, including flavour-linked toxicity [30,31,32], no studies have yet investigated their combined impact on airway cell survival. This suggests that dual use may lead to higher toxicity in the lungs than smoking.

Damage to the airways usually triggers increased cellular metabolism, leading to wound healing through cell proliferation, needed to replace damaged cells, a process which is hindered by cigarette smoke [33,34]. We investigated the cellular metabolism of MTT in single vs. dual exposures in 16HBE cells. We observed a decreased MTT conversion after exposure to two out of four flavours. However, one of the non-significant flavours was quite diluted to reduce it to a non-toxic level, so its impact at higher doses could not be assessed in this assay. Only one study has studied the effects of e-cigarettes on wound healing, and this study observed a negative impact on the gingival cells [35]. However, in this study, for two out of four flavours, dual exposure had a greater impact compared to CSE exposure.

Commercially available smoker NHBE cells allowed us to validate our results using both a Dual-User model—smokers who transition to both smoking and vaping, as well as a Switcher model—smokers who switch exclusively to e-cigarettes. Smoker bronchial epithelial cells showed reduced proliferative capacity in single exposure to three out of four flavours of EVE, whereas the fourth flavour was diluted to a non-toxic level, so its impact at higher concentrations could not be assessed in this assay. We also observed that dual exposure to CSE and EVE was greater than exposure to CSE for three out of four flavours. Interestingly, we also observed that the PGVG EVE alone also reduced the cellular proliferative potential, albeit to a lower extent than flavoured EVE, providing evidence that all e-liquids are likely to cause a negative impact, which can be exacerbated by the addition of flavours to varying extents. This study therefore provides the first early evidence that Dual Users may expose their airways to a higher toxicity risk.

In order to perform their primary function, monocytes need to be able to migrate to the site of infection and then differentiate into macrophages, so we assessed the impacts of CSE, EVE, and dual exposure on monocyte migration. First, we needed to confirm whether dual exposure caused increased cellular toxicity, so that we could be sure that cell death was not contributing to any declines. We did not observe any increased toxicity from dual exposure. However, the chocolate and banana flavours needed dilution to prevent toxicity. We then assessed whether EVE, CSE, or dual exposure affected monocyte differentiation and/or adhesion to ensure this was not reducing cell numbers. We found that CSE and two flavours needed diluting to avoid inducing differentiation, and thus adhesion to the plates, and falsely impacting the results. All four flavours and nicotine alone reduced monocyte migration. Dual exposure further reduced migration, which was also significantly reduced compared to CSE alone. Interestingly, PGVG-only EVE impacted THP-1 migration, possibly due to the coating of the cells with a viscous substance. Whilst there is little information on the effects of e-cigarettes on monocyte migration, a recent paper showed that THP-1 monocytes exposed to DHA had reduced migration [36]. We then confirmed these data in healthy non-smoker donor monocytes, with the same results. The existing literature shows conflicting information on the effects of CSE on monocyte migration, with some studies showing increased migration and others decreased migration, which may be due to methods of extraction, cell type, dose, or even different migration assay designs [37,38,39,40,41,42,43,44,45]. There are not any other studies yet investigating the impacts of e-cigarettes on monocyte migration, let alone dual exposure.

Once at the site of an infection, monocytes can differentiate into macrophages and phagocytose bacteria. Studies by us and others have shown that exposing immune cells to e-cigarette vapour causes reduced bacterial phagocytosis [6,7,8,23,46,47,48,49,50,51], with support from some mouse infection studies [24,51,52], but none have looked at dual exposure. This effect was most likely due to the decreased levels of phagocytic receptors TLR-2, TLR-4, CD44, CD36, and SR-A1 we observed on the surface of the cells [6,7,8,23]. This is the first study that demonstrates that exposure to both cigarette smoke and e-cigarette vapour causes a further reduction in the phagocytic capacity of macrophages.

Tissue-resident macrophages, including alveolar macrophages, act as first responders to infections and then release chemokines to recruit monocytes to the site of infection [53,54]. Thus, we assessed two key chemokines secreted by MDMs with single and dual exposure. We have previously shown that the secretion of the monocyte chemokine MCP-1 by THP-1 macrophages was decreased after exposure to CSE, nicotine, and some flavoured EVEs in single exposure [8], which was also found by Sinha et al. [55]. In mice, Wang et al. found increased MCP-1 and RANTES in bronchoalveolar lavage fluid (BALF) after exposure to vapour from unflavoured nicotine e-liquid [56]. The subsequent paper found that the inflammatory profile changes were often gender specific [57]. Moshensky et al. found no changes in the *RANTES* gene expression in nicotine-vaped mouse lung tissue, but only two flavours were tested [26]. MCP-1 increased in the BALF of mice exposed to unflavoured nicotine vapour [58]. In humans, one study showed that vapers had increased RANTES in airway cells [59], but another showed reduced RANTES in the sputum of vapers [60]. Hickman et al. found no change in MCP-1 in vapers’ sputum [61]. Cigarette smoke has been associated with reduced MCP-1 and RANTES in several studies [62,63,64,65,66,67,68], although there are conflicting studies as well [69,70,71,72]. No studies have yet looked at the effect of dual exposure on monocyte recruitment via chemokine production from macrophages. Our data show that dual exposure exacerbated the reduction in chemokine secretion.

We then assessed whether the neutrophil chemoattractant, IL-8 [73], was also reduced by single and dual exposures, but we found that this was actually increased with CSE, single EVE, and dual exposures, with greater increases for dual exposures. We have previously demonstrated increased IL-8 secretion by donor airway cells that was specific to certain flavours, but only a trend of increased IL-8 levels in saliva of vapers [7] (also seen by Perez et al. [74]), which we believe to be related to mixed flavour use in the vapers. This was supported by mixed results in the literature in studies which used a range of flavours [2,75,76,77,78]. IL-8 secretion by monocytes and macrophages, specifically, was also found to be related to certain flavours [79,80,81]. The literature on the role of cigarette smoke on macrophage IL-8 secretion consistently reported a positive association, similar to our findings [64,66,82,83,84], but, again, there are not any studies yet looking at dual exposure.

VOCs are widely recognised as toxic, with some being recognised as carcinogenic, and concentrations in workplaces are often regulated [85,86]. VOCs, due to their volatile oxidative nature, lead to oxidative stress and the formation of reactive oxygen species. When inhaled, they can cause pulmonary harm [87,88]. Whilst there are minimal data available in the literature on the direct impacts of VOC exposure without particulate matter, these few studies have shown it leads to the disruption of cytokine airway levels [89,90], including increased airway IL-8 [91,92,93].

VOCs in e-cigarettes have been shown to have a distinctly different VOC profile compared to cigarette smoke [19,94,95], not just a decrease in the VOCs present in cigarette smoke. This has been linked to the presence of flavourants in e-cigarettes vs. cigarettes [96]. Some previous studies have even shown that e-liquid is reactive, and that new products are formed between the flavouring chemicals and the glycol bases, even at room temperature [97,98,99]. Many e-liquids contain more than five different flavouring chemicals and as high as 50 [7,100]. We have previously assessed 10 different flavoured e-liquids and observed a correlation between the benzene ring-containing flavourants and impacts on macrophage phagocytosis and airway epithelial cell toxicity [7].

However, there has been little investigation into what may occur when the chemicals of both cigarette smoke and e-cigarette vapour are allowed to interact, i.e., in the lung lining fluid of a Dual User. We therefore allowed the soluble chemicals from cigarette smoke and e-cigarette vapour to interact and demonstrated that the interaction between the two can indeed lead to the alteration of the VOCs present in either e-cigarette vapour or cigarette smoke, as well as the formation of new ones, providing a plausible reason why dual exposure has been linked with poorer health outcomes.

These chemicals include aniline, which has been known to be toxic for almost 100 years and can be absorbed through skin or inhaled and can cause methemoglobinemia, or reduced ability of haemoglobin to carry oxygen, resulting in breathlessness [101]. Phosphoryl fluoride was also detected, a toxic fluoride gas produced in lithium battery fires [102], as well as pyrrole, which is associated with hepatotoxicity [103]. 3,5-dimethylphenol/3.5-DMP is known to be toxic to the waterways [104], and furfural has been shown to cause lung injury [105]. Whilst there is very little in the literature in this area, the study by Cirillo et al. showed that the level of carbonyls produced by e-liquids without nicotine was higher than that in those with nicotine. This was linked to the total concentration of chemicals in the e-liquid being lower and allowing more room for carbonyl creation [106].

We also observed a number of VOCs in single exposure extracts that were not present in the dual exposure, even in the samples frozen immediately after production. This suggests that the VOCs, which are by nature difficult to destroy without powerful catalytic compounds, were altered, perhaps by metabolism, oxidation, or thermal degradation. VOC by-products or intermediary VOCs formed from these processes are also often considered toxic. This may represent another possible pathway for the increased harm being observed in Dual Users.

Dual use if often considered harm reduction due to reduction in exposure to the chemicals and VOCs in cigarette smoke [107,108]. However, these data provide proof-of-concept evidence that using both tobacco cigarettes and e-cigarettes will expose users not only to the different sets of chemicals in cigarette smoke and e-cigarette vapour, but also to a likely new set of chemicals formed due to the interaction between the chemicals in cigarette smoke and e-cigarette vapour. This may be one of the reasons why increased harm is observed in dual use vs. smoking or vaping only. Further extensive investigation is warranted into the interaction of e-cigarette vapour with and without flavour or nicotine with cigarette smoke to further understand the additional harms dual use may present.

There are limitations to our study, in that we utilized in vitro or ex vivo systems, and that the airways exist as a complicated system involving many interacting cell types. We only tested four e-liquids, and there are currently thousands sold worldwide. As we used PG and VG sold by the vaping company as our PG:VG, the purity of these reagents could not be guaranteed. We only assessed the interaction of soluble chemicals. Assessment of VOCs and larger, dedicated mass spec studies are needed to further expand on our data. Due to the large number of cells needed for migration and phagocytosis assays, we were unable to assess these in Switcher and Dual-User models. We were also unable to assess impacts based on gender.

## 4. Materials and Methods

### 4.1. EVE and CSE Preparation

E-cigarette vapour extract (EVE) was produced using an EVOD-2 3.7 V, which uses a 1.5 Ω. dual coil, as previously described [8]. EVE from four flavoured e-liquids (banana, chocolate, mango, and tobacco), in a 50% Propylene Glycol:50% Vegetable Glycerine base (PG:VG), nicotine (at 18 mg/mL in PG:VG), and PG:VG were only used in this study. These flavours were initially tested as part of a panel of 10 flavours in previous studies, due to their popularity amongst non-smokers (sweet/fruit), smokers, and ex-smokers (tobacco). These four flavours were selected from the panel of 10 for future studies, as they represented flavours with low epithelial toxicity (tobacco and mango) and high epithelial toxicity (banana and chocolate) [7]. The control medium (C) was obtained by using the same apparatus to pass air through the culture medium for the same duration as e-cigarette use. Cigarette smoke extract (CSE) was prepared as previously described [8]. For migration assays, 5 cigarettes (250% CSE) or 60 × 3s puffs (120% EVE) per 10 mL of serum-free media were required to make combination treatments that could be added to the cells without diluting the EVE below 100%.

### 4.2. Cell Maintenance

The THP-1 monocytic cell line (ATCC, Manassas, VA, USA) was maintained in RPMI with 10% FCS and differentiated into macrophages, as previously described [23]. Experiments were carried out within 10 passages of each other. Cells were routinely screened for mycoplasma.

The 16HBE14o- airway epithelial cell line (16HBE, #SCC150 from Sigma Aldrich, St. Louis, MO, USA) was maintained in a MEM medium supplemented with L-glutamine (2 mM), penicillin (12 µg/mL), gentamycin (16 µg/mL), and 10% FCS. Experiments were carried out within 10 passages of each other. Cells were routinely screened for mycoplasma.

Normal human bronchial epithelial cells from healthy patients with a history of cigarette smoking (SNHBE; CC-2540, Lonza, Durham, NC, USA) were cultured as per manufacturer’s instructions in basal epithelial growth medium (BEGM) and used within 3 passages.

### 4.3. Isolation of Monocytes and Monocyte-Derived Macrophages

Monocytes were collected from non-smokers with no history of chronic respiratory disease. Written, informed consent was obtained from all participants, and ethics approval was granted by the Royal Adelaide Hospital HREC for this research. Peripheral blood mononuclear cells (PBMCs) were isolated as previously described [109] and seeded for 45 min to allow monocyte adherence. Non-adherent cells were rinsed off. Monocytes were either differentiated into monocyte-derived macrophages (MDMs), as previously described [109], or lifted by pipetting after 10 min in warm 10× TrypLE Select (Life Technologies, Carlsbad, CA, USA) for seeding into assay plates.

### 4.4. MTT Assay

Both 16HBE and smoker NHBE cells were seeded, in triplicate, in 200 µL of growth medium at 1 × 10^4^ cells/well in 96-well plates and allowed to adhere overnight. The growth medium was replaced with 100 µL of treatment for 24 h with 10 µL of 5 mg/mL MTT (3-(4,5-dimethylthiazol-2-yl)-2,5-diphenyl tetrazolium bromide; Sigma Aldrich), in PBS, added for the final 4 h of incubation. Treatment media were carefully removed and the formazan crystals were solubilised with 50 µL of dimethyl sulfoxide (Sigma Aldrich) before absorbance at 570 nm was measured.

### 4.5. Phagocytosis Assay

Non-typeable *Haemophilus influenzae* (NTHi) were prepared, and the phagocytosis assay was performed with a 50:1 target-to-macrophage ratio, as previously described [8]. Treated macrophages that were not exposed to targets were used as a gating control, as previously demonstrated [8], and phagocytosis was assessed by collecting events on a FACSCanto II with FACS DIVA 7.0 (BD Biosciences, Milpitas, CA, USA).

### 4.6. Migration Assay

THP-1 monocytes, at a concentration of 1 × 10^6^ cells/mL, were labelled with 0.4 µM Calcein AM for 30 min at room temperature in serum-free media. Excess Calcein was removed by centrifugation 150× *g* for 5 min. The cell pellet was resuspended twice in serum-free media and centrifuged. A mixture of 120% EVE and 250% CSE was used to allow 100% EVE + 5% CSE treatment. Cells were treated under serum-free conditions for 22.5 h before 50,000 cells were added to the triplicate wells of a ChemoTX™ chemotaxis plate (5 µm pore, #111-5, Neuroprobe, Gaithersburg, MD, USA), with 10% FCS + 100 ng/mL IL-2 in the bottom chambers as a chemoattractant. The top filter was wiped with a cotton bud and then gently rinsed with PBS. The plate, with the filter still attached, was centrifuged at 150× *g* for 5 min and then read at 490/520 nm. Pre-treatment with 10 µM Cytochalasin D was used as a negative control, and the cells added directly to the bottom well served as a positive control, to validate the assay method.

### 4.7. Differentiation Assay

To assess whether EVE and CSE treatments induced spontaneous THP-1 monocyte differentiation, as measured by induction of adhesion, 50,000 THP-1 monocytes were seeded and treated as per the migration assay for 24 h. Differentiation was induced by 45 µM phorbol 12-myristate 13-acetate (PMA) as a positive control. Non-adherent cells were gently removed and adherent cells gently rinsed once with sterile PBS. Adherent cells were stained with 1% Crystal Violet in methanol for 30 min and washed with water 3 times. Plates were air dried for 5 min before adding 95% ethanol and placed on a shaker for 15 min to dissolve the crystal violet. Absorbance at 570 nm was measured.

### 4.8. LDH Assay

For epithelial cell toxicity analysis, the supernatant was collected from MTT assay plates, and for THP-1 monocytes, the supernatant was collected from differentiation assay plates. Supernatants were analysed for lactate dehydrogenase (LDH) activity as previously described [8]. The assay was performed as per manufacturer’s instructions (Roche, Basel, Switzerland). In a separate well, Tween-20 was added at 2% *v*/*v*, mixed and incubated for 5 min to lyse cells as a 100% control of maximal LDH release.

### 4.9. Cytokine Analysis

The supernatant was collected from treated MDMs being used for phagocytosis assays. The analysis of MDM-secreted cytokines via cytometric bead array (CBA, BD Biosciences) was performed as per the manufacturer guidelines for cell culture supernatants. CBA was analysed on a FACSCantoII with FCAP array software v3.0. Human soluble protein flex sets were used. The targeted cytokines were RANTES, IL-8, MCP-1, IP-10, and MIG. The supernatant was tested at a 1:4 dilution.

### 4.10. Mass Spectrometry of E-Cigarette Vapour, Cigarette Smoker, and Dual Extracts

EVE, CSE, and dual extracts were externally analysed by Envirolab Services Pty Ltd. (Perth, Australia) for volatile organic chemical (VOC) markers using gas chromatography mass spectrometry (Agilent Technologies, Mulgrave, Australia). The extract samples were diluted with ultra-high-purity water and spiked with relevant internal standards prior to analysis using a Restek Rxi-624Sil MS GC column (Restek Corporation, Bellefonte, PA, USA), with specifications of 20 m × 0.18 mm, 1.0 µm film thickness, fitted with a 1 m × 0.32 mm deactivated guard. The inlet temperature was set at 250 °C and samples (0.5 µL) were injected with a 5:1 split. Helium was used as the carrier gas, and the GC temperature program was as follows: 60 °C for 8 min; 2 °C/min to 90 °C; 5 °C/min to 150 °C; 20 °C/min to 230 °C; then hold for 2 min. The MS detector was operated at 300 °C with a solvent delay of 3 min across a *m*/*z* scan of 20–550.

### 4.11. Statistical Analysis

The data are presented as mean ± SEM for cell line work or median and individual data points for ex vivo work. The Kruskal–Wallis non-parametric ANOVA with the Mann–Whitney U test was employed for statistical analysis for cell line work. Friedman’s non-parametric test with the Wilcoxon signed ranks pairwise test was performed for analysis of the primary cell work. Differences from control of *p* < 0.05 were considered significant. All statistical tests were performed using Graphpad Prism 9.

## 5. Conclusions

These data, combined with previous work by others, highlight the fact that dual use of tobacco cigarettes and e-cigarettes may place Dual Users at risk of increased harm instead of the marketed concept of harm reduction. Our data suggest Dual Users may be at greater risk from infections than smokers by way of reduced phagocytosis of bacteria, reduced monocyte recruitment signals and, consequently, reduced monocyte migration towards the infection site. We provide one possible reason for this: the effects of altered chemicals created by the interaction of reactive chemicals in cigarette smoke with the chemicals in e-cigarette vapour, and vice versa, which could occur in the lungs of Dual Users. When one considers that dual use exposes the user to an increased number of chemicals compared to smoking alone, along with exposure to chemicals formed by the interaction between cigarette smoke and e-cigarette vapour, dual use should not be considered as a form of harm reduction.

## Figures and Tables

**Table 1:** Significance values for comparisons between single and dual exposure treatments on the proliferative potential of SNHBE cells.

Treatment	Vs. Control	Vs. CSE	Single vs. Dual	Vs. PGVG	Vs. 25% PGVG	Vs. Dual PGVG	Vs. Dual 25% PGVG
**CSE**	NS	-	-	-	-	-	-
**Nicotine**	NS	NS	NS	0.0005	-	-	-
**Dual Nicotine**	NS	NS		-	-	0.0210	-
**Tobacco**	0.0049	0.0269	0.0161	NS	-	-	-
**Dual Tobacco**	0.0024	0.0024		-	-	0.0269	-
**Mango**	0.0269	0.0425	0.0068	NS	-	-	-
**Dual Mango**	0.0010	0.0029		-	-	0.0024	-
**PGVG**	0.0068	0.0425	NS	-	-	-	-
**Dual PGVG**	0.0010	0.0049		-	NS	-	-
**Banana**	NS	NS	0.0068	-	0.0425	-	-
**Dual Banana**	0.0269	0.0342		-	-	-	NS
**Chocolate**	NS	0.0122	0.0425	-	0.0010	-	-
**Dual Chocolate**	NS	NS		-	-	-	NS
**25% PGVG**	0.0342	NS	NS	-	-	-	-
**Dual 25% PGVG**	0.0049	0.0342		-	-	-	NS

Nicotine, mango, and tobacco were used at 100% whilst banana and chocolate were used at 25%. Values for *p* < 0.05 are presented. NS = not significant. -, comparison not applicable.

**Table 2:** Significance between comparisons of single and dual exposures on THP-1 monocyte migration.

Treatment	Vs. Control	Vs. CSE	Single vs. Dual	Vs. PGVG	Vs. 25% PGVG	Vs. Dual PGVG	Vs. Dual 25% PGVG
**CSE**	0.0078	-	-	-	-	-	-
**Nicotine**	0.0078	NS	0.0078	0.0156	-	-	-
**Dual Nicotine**	0.0078	0.0078		-	-	NS	-
**Tobacco**	0.0156	0.0156	0.0313	0.0156	-	-	-
**Dual Tobacco**	0.0313	0.0313		-	-	NS	-
**Mango**	0.0078	NS	0.0078	0.0156	-	-	-
**Dual Mango**	0.0078	0.0078		-	-	NS	-
**PGVG**	0.0078	NS	0.0078	-	-	-	-
**Dual PGVG**	0.0078	0.0156		-	0.0078	-	-
**Banana**	0.0078	NS	0.0078	-	0.0078	-	-
**Dual Banana**	0.0078	0.0078		-	-	-	0.0078
**Chocolate**	0.0078	NS	0.0078	-	0.0078	-	-
**Dual Chocolate**	0.0078	0.0078		-	-	-	0.0078
**25% PGVG**	NS	0.0078	0.0078	-	-	-	-
**Dual 25% PGVG**	0.0078	NS		-	-	-	0.0078

Nicotine, mango, and tobacco were used at 100%, whilst banana and chocolate were used at 25%. *p* < 0.05 values presented. NS = not significant. -, comparison not applicable.

**Table 3:** Significant differences between comparisons of single and dual exposures on healthy non-smoker monocyte migration.

Treatment	Vs. Control	Vs. CSE	Single vs. Dual	Vs. PGVG	Vs. 25% PGVG	Vs. Dual PGVG	Vs. Dual 25% PGVG
**CSE**	0.002	-	-	-	-	-	-
**Nicotine**	0.002	NS	0.002	NS	-	-	-
**Dual Nicotine**	0.002	0.002		-	-	0.0195	-
**Tobacco**	0.002	NS	0.002	NS	-	-	-
**Dual Tobacco**	0.002	0.002		-	-	0.0273	-
**Mango**	0.002	NS	0.002	NS	-	-	-
**Dual Mango**	0.002	0.002		-	-	0.0195	-
**PGVG**	0.002	NS	NS	-	-	-	-
**Dual PGVG**	NS	0.002		-	NS	-	-
**Banana**	0.002	NS	0.002	-	0.002	-	-
**Dual Banana**	0.002	0.002		-	-	-	0.002
**Chocolate**	0.0371	0.0039	0.002	-	0.0273	-	-
**Dual Chocolate**	0.002	0.0137		-	-	-	0.0098
**25% PGVG**	NS	0.002	NS	-	-	-	-
**Dual 25% PGVG**	0.002	NS		-	-	-	NS

Nicotine, mango, and tobacco were used at 100%, whilst banana and chocolate were used at 25%. *p* < 0.05 for all the values presented. NS = not significant. -, comparison not applicable.

**Table 4:** Mass spectrometry analysis of VOCs in single- and dual-extract samples frozen immediately after collection.

RT	Chemical	CSE	CHOC	CSE into CHOC	CHOC into CSE
3.32	2,2-dimethyl-propanal	DETECTED		REDUCED DETECTION	NO LONGER DETECTED
3.35	3-propoxy-1-propene				
3.90	2-Pentanone	DETECTED		NO LONGER DETECTED	NO LONGER DETECTED
3.91	Allyl pentyl ester oxalic acid	DETECTED		REDUCED DETECTION	NO LONGER DETECTED
4.03	Acetic acid ethenyl ester	DETECTED		NO LONGER DETECTED	NO LONGER DETECTED
5.46	Pyridine	DETECTED		STILL DETECTED	STILL DETECTED
7.72	Pyrrole	DETECTED		STILL DETECTED	STILL DETECTED
8.13	Cyclopentanone	DETECTED		STILL DETECTED	NO LONGER DETECTED
8.58	Isopropyl alcohol				
8.73	Propylene glycol		DETECTED	STILL DETECTED	STILL DETECTED
9.07	Methylpyrazine	DETECTED		NO LONGER DETECTED	NO LONGER DETECTED
11.23	2-Cyclopenten-1-one				
11.29	Furfural				
11.29	2-oxo-3-cyclopentene-1-acetaldehyde	DETECTED		NO LONGER DETECTED	STILL DETECTED
11.85	3-methylpyridine	DETECTED		NO LONGER DETECTED	STILL DETECTED
11.87	Aniline			NEW	
11.93	Diacetate 1,1-ethanediol				
14.80	2,5-dimethylpyrazine/2,6-dimethylpyrazine/2,5-Dimethylpyrimidine	DETECTED	DETECTED	STILL DETECTED	STILL DETECTED
15.81	2-methyl-2-cyclopenten-1-one	DETECTED		STILL DETECTED	STILL DETECTED
19.74	2-ethenyl-pyridine	DETECTED		NO LONGER DETECTED	NO LONGER DETECTED
21.52	Trimethylpyrazine		DETECTED	STILL DETECTED	STILL DETECTED
21.62	3-methyl-2-cyclopenten-1-one	DETECTED		NO LONGER DETECTED	NO LONGER DETECTED
22.09	2,3-dihydroxy-propanal				
22.10	Methyl alcohol				
22.12	Methylglyoxal				
22.13	1,3-dihydroxy-2-Propanone	DETECTED	DETECTED	STILL DETECTED	STILL DETECTED
22.70	1-(3H-Imidazol-4-yl)-ethanone	DETECTED		REDUCED DETECTION	STILL DETECTED
26.03	3,4-dimethyl-2-cyclopenten-1-one/2,3-dimethyl-2-cyclopenten-1-one	DETECTED		STILL DETECTED	
26.75	Phenol/Vinyl furan	DETECTED		STILL DETECTED	STILL DETECTED
26.84	2,5,6,7-tetrahydro-3H-Cyclopenta[c]pyridazin-3-one/3-methylene-2-oxo-cyclohexanecarboxylic acid, methyl ester		DETECTED	STILL DETECTED	NO LONGER DETECTED
26.85	1-ethoxy-2-methylbenzene				NEW
27.00	Glycerin	DETECTED	DETECTED	STILL DETECTED	STILL DETECTED
28.58	2-Acetyl-5-methylfuran/2-methoxyphenol/Mequinol	DETECTED	DETECTED	STILL DETECTED	STILL DETECTED
29.36	2-methylphenol	DETECTED		STILL DETECTED	STILL DETECTED
30.56	4-methylphenol	DETECTED		STILL DETECTED	STILL DETECTED
30.78	N-methyl-1-Octanamine				
30.80	Acetaldehyde				
32.66	3,5-dimethylphenol				
32.67	1-methoxy-4-methyl-benzene	DETECTED		NO LONGER DETECTED	NO LONGER DETECTED
33.57	3-pyridinol	DETECTED		STILL DETECTED	STILL DETECTED
33.59	3-pyridinamine				NEW
33.76	4-ethylphenol	DETECTED		REDUCED DETECTION	NO LONGER DETECTED
35.88	Phosphoryl fluoride				NEW
36.82	3-(1-methyl-2-pyrrolidinyl)pyridine	DETECTED		STILL DETECTED	STILL DETECTED
36.96	Indole/m-Aminophenylacetylene	DETECTED		STILL DETECTED	STILL DETECTED
38.08	3-(3,4-dihydro-2H-pyrrol-5-yl)-pyridine	DETECTED		STILL DETECTED	STILL DETECTED
38.12	Vanillin/2-hydroxy-4-methoxy-benzaldehyde/3-hydroxy-4-methoxy-benzaldehyde		DETECTED	STILL DETECTED	STILL DETECTED
38.36	N-methyl-benzeneacetamide				NEW
38.38	Benzeneacetamide				
38.64	3-methyl-1-phenyl-1H-pyrazole				
38.65	2,2′-Bipyrazine			NEW	
39.23	2,3′-Dipyridyl	DETECTED		STILL DETECTED	STILL DETECTED

STILL DETECTED = detected in single extracts and also in dual extracts. NO LONGER DETECTED = detected in single extracts and no longer detected in dual extracts. REDUCED DETECTION = full detection in single extracts, only trace detection in dual extracts. NEW = only detected in dual extracts and not detected in single extracts.

**Table 5:** Mass spectrometry analysis of VOCs in single- and dual-extract samples incubated at 37 °C with 5% CO_2_ for 24 h immediately after collection.

RT	Chemical	CSE	CHOC	CSE into CHOC	CHOC into CSE
3.32	2,2-dimethyl-propanal				
3.35	3-propoxy-1-propene				
3.90	2-Pentanone				
3.91	Allyl pentyl ester oxalic acid				
4.03	Acetic acid ethenyl ester				
5.46	Pyridine	DETECTED		STILL DETECTED	STILL DETECTED
7.72	Pyrrole			NEW	NEW
8.13	Cyclopentanone			NEW	
8.58	Isopropyl alcohol				
8.73	Propylene glycol		DETECTED	STILL DETECTED	STILL DETECTED
9.07	Methylpyrazine	DETECTED		NO LONGER DETECTED	NO LONGER DETECTED
11.23	2-Cyclopenten-1-one				
11.29	Furfural				
11.29	2-oxo-3-cyclopentene-1-acetaldehyde				
11.85	3-methylpyridine				
11.87	Aniline				
11.93	Diacetate 1,1-ethanediol				
14.80	2,5-dimethylpyrazine/2,6-dimethylpyrazine/2,5-Dimethylpyrimidine	DETECTED		STILL DETECTED	STILL DETECTED
15.81	2-methyl-2-cyclopenten-1-one	DETECTED		STILL DETECTED	STILL DETECTED
19.74	2-ethenyl-pyridine				
21.52	Trimethylpyrazine				
21.62	3-methyl-2-cyclopenten-1-one			NEW	NEW
22.09	2,3-dihydroxy-propanal				
22.10	Methyl alcohol	DETECTED		NO LONGER DETECTED	NO LONGER DETECTED
22.12	Methylglyoxal		DETECTED	NO LONGER DETECTED	NO LONGER DETECTED
22.13	1,3-dihydroxy-2-Propanone	DETECTED		STILL DETECTED	STILL DETECTED
22.70	1-(3H-Imidazol-4-yl)-ethanone				
26.03	3,4-dimethyl-2-cyclopenten-1-one/2,3-dimethyl-2-cyclopenten-1-one	DETECTED		STILL DETECTED	NO LONGER DETECTED
26.75	Phenol/Vinyl furan	DETECTED		STILL DETECTED	STILL DETECTED
26.84	2,5,6,7-tetrahydro-3H-Cyclopenta[c]pyridazin-3-one/3-methylene-2-oxo-cyclohexanecarboxylic acid, methyl ester				
26.85	1-ethoxy-2-methylbenzene				
27.00	Glycerin	DETECTED	DETECTED	STILL DETECTED	STILL DETECTED
28.58	2-Acetyl-5-methylfuran/2-methoxyphenol/Mequinol			STILL DETECTED	STILL DETECTED
29.36	2-methylphenol	DETECTED		STILL DETECTED	STILL DETECTED
30.56	4-methylphenol	DETECTED		STILL DETECTED	STILL DETECTED
30.78	N-methyl-1-Octanamine				
30.80	Acetaldehyde				
32.66	3,5-dimethylphenol				
32.67	1-methoxy-4-methyl-benzene				
33.57	3-pyridinol	DETECTED		NO LONGER DETECTED	STILL DETECTED
33.59	3-pyridinamine				
33.76	4-ethylphenol				
35.88	Phosphoryl fluoride				
36.82	3-(1-methyl-2-pyrrolidinyl)pyridine	DETECTED	DETECTED	STILL DETECTED	STILL DETECTED
36.96	Indole/m-Aminophenylacetylene	DETECTED		STILL DETECTED	STILL DETECTED
38.08	3-(3,4-dihydro-2H-pyrrol-5-yl)-pyridine	DETECTED		STILL DETECTED	STILL DETECTED
38.12	Vanillin/2-hydroxy-4-methoxy-benzaldehyde/3-hydroxy-4-methoxy-benzaldehyde				
38.36	N-methyl-benzeneacetamide				
38.38	Benzeneacetamide			STILL DETECTED	STILL DETECTED
38.64	3-methyl-1-phenyl-1H-pyrazole				
38.65	2,2′-Bipyrazine				
39.23	2,3′-Dipyridyl	DETECTED		STILL DETECTED	STILL DETECTED

STILL DETECTED = detected in single extracts and also in dual extracts. NO LONGER DETECTED = detected in single extracts and no longer detected in dual extracts. NEW = only detected in dual extracts and not detected in single extracts.

## Data Availability

Data is contained within the article and Supplementary Materials.

## Supplementary Materials

The following supporting information can be downloaded at: https://www.mdpi.com/article/10.3390/ijms25116071/s1.

## References

[1] Bozier J., Chivers E.K., Chapman D.G., Larcombe A.N., Bastian N.A., Masso-Silva J.A., Byun M.K., McDonald C.F., Crotty-Alexander L.E., Ween M.P. (2020). The Evolving Landscape of e-Cigarettes: A Systematic Review of Recent Evidence. Chest.

[2] Lerner C.A., Sundar I.K., Yao H., Gerloff J., Ossip D.J., McIntosh S., Robinson R., Rahman I. (2015). Vapors produced by electronic cigarettes and e-juices with flavorings induce toxicity, oxidative stress, and inflammatory response in lung epithelial cells and in mouse lung. PLoS ONE.

[3] Crotty Alexander L.E., Drummond C.A., Hepokoski M., Mathew D.P., Moshensky A., Willeford A., Das S., Singh P., Yong Z., Lee J.H. (2018). Chronic Inhalation of E-Cigarette Vapor Containing Nicotine Disrupts Airway Barrier Function and Induces Systemic Inflammation and Multi-Organ Fibrosis in Mice. Am. J. Physiol. Regul. Integr. Comp. Physiol..

[4] National Academies of Sciences, Engineering, and Medicine (2018). Public Health Consequences of E-Cigarettes.

[5] Banks E., Yazidjoglou A., Brown S., Nguyen M., Martin M., Beckwith K., Daluwatta A., Campbell S., Joshy G. (2023). Electronic cigarettes and health outcomes: Umbrella and systematic review of the global evidence. Med. J. Aust..

[6] Ween M.P., Hamon R., Macowan M.G., Thredgold L., Reynolds P.N., Hodge S.J. (2020). Effects of E-cigarette E-liquid components on bronchial epithelial cells: Demonstration of dysfunctional efferocytosis. Respirology.

[7] Ween M.P., Moshensky A., Thredgold L.L., Bastian N.A., Hamon R., Badiei A., Nguyen P.T., Herewane K., Jersmann H., Bojanowski C.M. (2020). E-cigarettes and health risks: More to the flavour than just the name. Am. J. Physiol. Lung Cell. Mol. Physiol..

[8] Ween M.P., Whittall J.J., Hamon R., Reynolds P.N., Hodge S.J. (2017). Phagocytosis and Inflammation: Exploring the effects of the components of E-cigarette vapor on macrophages. Physiol. Rep..

[9] AIHW (2020). National Drug Strategy Household Survey 2019.

[10] Owusu D., Huang J., Weaver S.R., Pechacek T.F., Ashley D.L., Nayak P., Eriksen M.P. (2019). Patterns and trends of dual use of e-cigarettes and cigarettes among U.S. adults, 2015–2018. Prev. Med. Rep..

[11] Coleman S.R.M., Piper M.E., Byron M.J., Bold K.W. (2022). Dual Use of Combustible Cigarettes and E-cigarettes: A Narrative Review of Current Evidence. Curr. Addict. Rep..

[12] Osei A.D., Mirbolouk M., Orimoloye O.A., Dzaye O., Uddin S.I., Benjamin E.J., Hall M.E., DeFilippis A.P., Stokes A., Bhatnagar A. (2019). Association between e-cigarette use and cardiovascular disease among never and current combustible-cigarette smokers. Am. J. Med..

[13] Goniewicz M.L., Smith D.M., Edwards K.C., Blount B.C., Caldwell K.L., Feng J., Wang L., Christensen C., Ambrose B., Borek N. (2018). Comparison of nicotine and toxicant exposure in users of electronic cigarettes and combustible cigarettes. JAMA Netw. Open.

[14] Wang J.B., Olgin J.E., Nah G., Vittinghoff E., Cataldo J.K., Pletcher M.J., Marcus G.M. (2018). Cigarette and e-cigarette dual use and risk of cardiopulmonary symptoms in the Health eHeart Study. PLoS ONE.

[15] Jackson S.E., Shahab L., West R., Brown J. (2020). Associations between dual use of e-cigarettes and smoking cessation: A prospective study of smokers in England. Addict. Behav..

[16] Lee M.S., LeBouf R.F., Son Y.S., Koutrakis P., Christiani D.C. (2017). Nicotine, aerosol particles, carbonyls and volatile organic compounds in tobacco- and menthol-flavored e-cigarettes. Environ. Health.

[17] Larcombe A., Allard S., Pringle P., Mead-Hunter R., Anderson N., Mullins B. (2022). Chemical analysis of fresh and aged Australian e-cigarette liquids. Med. J. Aust..

[18] Pennings J.L.A., Cremers J., Becker M.J.A., Klerx W.N.M., Talhout R. (2020). Aldehyde and Volatile Organic Compound Yields in Commercial Cigarette Mainstream Smoke Are Mutually Related and Depend on the Sugar and Humectant Content in Tobacco. Nicotine Tob. Res..

[19] Berenguer C., Pereira J.A.M., Câmara J.S. (2021). Fingerprinting the volatile profile of traditional tobacco and e-cigarettes: A comparative study. Microchem. J..

[20] Kuehl P.J., McDonald J.D., Weber D.T., Khlystov A., Nystoriak M.A., Conklin D.J. (2022). Composition of aerosols from thermal degradation of flavors used in ENDS and tobacco products. Inhal. Toxicol..

[21] Lucchiari C., Masiero M., Mazzocco K., Veronesi G., Maisonneuve P., Jemos C., Sale E.O., Spina S., Bertolotti R., Busacchio D. (2022). Nicotine-Free E-Cigarettes Might Promote Tobacco Smoking Reduction Better Than Nicotine Delivery Devices: Results of a Double-Blind Randomized Controlled Trial at 1 Year. Curr. Oncol..

[22] Hajek P., Phillips-Waller A., Przulj D., Pesola F., Myers Smith K., Bisal N., Li J., Parrott S., Sasieni P., Dawkins L. (2019). A Randomized Trial of E-Cigarettes versus Nicotine-Replacement Therapy. N. Engl. J. Med..

[23] Ween M., Ahern J., Carroll A., Hodge G., Pizzutto S., Jersmann H., Reynolds P., Hodge S. (2015). A small volume technique to examine and compare alveolar macrophage phagocytosis of apoptotic cells and non typeable Haemophilus influenzae (NTHi). J. Immunol. Methods.

[24] Sharma A., Lee J., Fonseca A.G., Moshensky A., Kothari T., Sayed I.M., Ibeawuchi S.R., Pranadinata R.F., Ear J., Sahoo D. (2021). E-cigarettes compromise the gut barrier and trigger inflammation. iScience.

[25] Boddu S.A., Bojanowski C.M., Lam M.T., Advani I.N., Scholten E.L., Sun X., Montgrain P., Malhotra A., Jain S., Alexander L.E.C. (2019). Use of Electronic Cigarettes with Conventional Tobacco Is Associated with Decreased Sleep Quality in Women. Am. J. Respir. Crit. Care Med..

[26] Moshensky A., Brand C.S., Alhaddad H., Shin J., Masso-Silva J.A., Advani I., Gunge D., Sharma A., Mehta S., Jahan A. (2022). Effects of mango and mint pod-based e-cigarette aerosol inhalation on inflammatory states of the brain, lung, heart, and colon in mice. eLife.

[27] Tang M.S., Wu X.R., Lee H.W., Xia Y., Deng F.M., Moreira A.L., Chen L.C., Huang W.C., Lepor H. (2019). Electronic-cigarette smoke induces lung adenocarcinoma and bladder urothelial hyperplasia in mice. Proc. Natl. Acad. Sci. USA.

[28] Lee H.W., Park S.H., Weng M.W., Wang H.T., Huang W.C., Lepor H., Wu X.R., Chen L.C., Tang M.S. (2018). E-cigarette smoke damages DNA and reduces repair activity in mouse lung, heart, and bladder as well as in human lung and bladder cells. Proc. Natl. Acad. Sci. USA.

[29] Bahl V., Lin S., Xu N., Davis B., Wang Y.H., Talbot P. (2012). Comparison of electronic cigarette refill fluid cytotoxicity using embryonic and adult models. Reprod. Toxicol..

[30] Kosmider L., Sobczak A., Prokopowicz A., Kurek J., Zaciera M., Knysak J., Smith D., Goniewicz M.L. (2016). Cherry-flavoured electronic cigarettes expose users to the inhalation irritant, benzaldehyde. Thorax.

[31] Leslie L.J., Vasanthi Bathrinarayanan P., Jackson P., Mabiala Ma Muanda J.A., Pallett R., Stillman C.J.P., Marshall L.J. (2017). A comparative study of electronic cigarette vapor extracts on airway-related cell lines in vitro. Inhal. Toxicol..

[32] Clapp P.W., Lavrich K.S., van Heusden C.A., Lazarowski E.R., Carson J.L., Jaspers I. (2019). Cinnamaldehyde in flavored e-cigarette liquids temporarily suppresses bronchial epithelial cell ciliary motility by dysregulation of mitochondrial function. Am. J. Physiol. Lung Cell. Mol. Physiol..

[33] Di Vincenzo S., Ninaber D.K., Cipollina C., Ferraro M., Hiemstra P.S., Pace E. (2022). Cigarette Smoke Impairs Airway Epithelial Wound Repair: Role of Modulation of Epithelial-Mesenchymal Transition Processes and Notch-1 Signaling. Antioxidants.

[34] Heijink I.H., Brandenburg S.M., Postma D.S., van Oosterhout A.J. (2012). Cigarette smoke impairs airway epithelial barrier function and cell-cell contact recovery. Eur. Respir. J..

[35] Alanazi H., Park H.J., Chakir J., Semlali A., Rouabhia M. (2018). Comparative study of the effects of cigarette smoke and electronic cigarettes on human gingival fibroblast proliferation, migration and apoptosis. Food Chem. Toxicol. An. Int. J. Publ. Br. Ind. Biol. Res. Assoc..

[36] Rodway L.A., Pauls S.D., Pascoe C.D., Aukema H.M., Taylor C.G., Zahradka P. (2023). Distinct effects of alpha-linolenic acid and docosahexaenoic acid on the expression of genes related to cholesterol metabolism and the response to infection in THP-1 monocytes and immune cells of obese humans. Biomed. Pharmacother..

[37] Kay A.B., McVie J.G. (1977). Monocyte chemotaxis in bronchial carcinoma and cigarette smokers. Br. J. Cancer.

[38] Shen Y., Rattan V., Sultana C., Kalra V.K. (1996). Cigarette smoke condensate-induced adhesion molecule expression and transendothelial migration of monocytes. Am. J. Physiol..

[39] Shapiro S.D., Goldstein N.M., Houghton A.M., Kobayashi D.K., Kelley D., Belaaouaj A. (2003). Neutrophil elastase contributes to cigarette smoke-induced emphysema in mice. Am. J. Pathol..

[40] Pott G.B., Tsurudome M., Bui J., Banfield C., Hourieh S., Pratap H., Goalstone M.L. (2017). VCAM-1 Mediates Cigarette Smoke Extract Enhancement of Monocyte Adhesion to Human Carotid Vascular Endothelial Cells. Med. Res. Arch..

[41] Mulligan J.K., O’Connell B.P., Pasquini W., Mulligan R.M., Smith S., Soler Z.M., Atkinson C., Schlosser R.J. (2017). Impact of tobacco smoke on upper airway dendritic cell accumulation and regulation by sinonasal epithelial cells. Int. Forum Allergy Rhinol..

[42] Stadler N., Eggermann J., Voo S., Kranz A., Waltenberger J. (2007). Smoking-induced monocyte dysfunction is reversed by vitamin C supplementation in vivo. Arterioscler. Thromb. Vasc. Biol..

[43] Ravi A.K., Plumb J., Gaskell R., Mason S., Broome C.S., Booth G., Catley M., Vestbo J., Singh D. (2017). COPD monocytes demonstrate impaired migratory ability. Respir. Res..

[44] Koyama S., Rennard S.I., Daughton D., Shoji S., Robbins R.A. (1991). Bronchoalveolar lavage fluid obtained from smokers exhibits increased monocyte chemokinetic activity. J. Appl. Physiol..

[45] Xu X., Wang H., Wang Z., Xiao W. (2009). Plasminogen activator inhibitor-1 promotes inflammatory process induced by cigarette smoke extraction or lipopolysaccharides in alveolar epithelial cells. Exp. Lung Res..

[46] Serpa G.L., Renton N.D., Lee N., Crane M.J., Jamieson A.M. (2020). Electronic Nicotine Delivery System Aerosol-induced Cell Death and Dysfunction in Macrophages and Lung Epithelial Cells. Am. J. Respir. Cell Mol. Biol..

[47] Scott A., Lugg S.T., Aldridge K., Lewis K.E., Bowden A., Mahida R.Y., Grudzinska F.S., Dosanjh D., Parekh D., Foronjy R. (2018). Pro-inflammatory effects of e-cigarette vapour condensate on human alveolar macrophages. Thorax.

[48] Hickman E., Herrera C.A., Jaspers I. (2019). Common E-Cigarette Flavoring Chemicals Impair Neutrophil Phagocytosis and Oxidative Burst. Chem. Res. Toxicol..

[49] Gomez A.C., Rodriguez-Fernandez P., Villar-Hernandez R., Gibert I., Muriel-Moreno B., Lacoma A., Prat-Aymerich C., Dominguez J. (2020). E-cigarettes: Effects in phagocytosis and cytokines response against *Mycobacterium tuberculosis*. PLoS ONE.

[50] Corriden R., Moshensky A., Bojanowski C.M., Meier A., Chien J., Nelson R.K., Crotty Alexander L.E. (2020). E-cigarette use increases susceptibility to bacterial infection by impairment of human neutrophil chemotaxis, phagocytosis, and NET formation. Am. J. Physiol. Cell Physiol..

[51] Hwang J.H., Lyes M., Sladewski K., Enany S., McEachern E., Mathew D.P., Das S., Moshensky A., Bapat S., Pride D.T. (2016). Electronic cigarette inhalation alters innate immunity and airway cytokines while increasing the virulence of colonizing bacteria. J. Mol. Med..

[52] Sussan T.E., Gajghate S., Thimmulappa R.K., Ma J., Kim J.H., Sudini K., Consolini N., Cormier S.A., Lomnicki S., Hasan F. (2015). Exposure to electronic cigarettes impairs pulmonary anti-bacterial and anti-viral defences in a mouse model. PLoS ONE.

[53] Shi C., Pamer E.G. (2011). Monocyte recruitment during infection and inflammation. Nat. Rev. Immunol..

[54] Chiu S., Bharat A. (2016). Role of monocytes and macrophages in regulating immune response following lung transplantation. Curr. Opin. Organ. Transplant..

[55] Sinha I., Goel R., Bitzer Z.T., Trushin N., Liao J., Sinha R. (2022). Evaluating electronic cigarette cytotoxicity and inflammatory responses in vitro. Tob. Induc. Dis..

[56] Wang Q., Sundar I.K., Li D., Lucas J.H., Muthumalage T., McDonough S.R., Rahman I. (2020). E-cigarette-induced pulmonary inflammation and dysregulated repair are mediated by nAChR alpha7 receptor: Role of nAChR alpha7 in SARS-CoV-2 COVID-19 ACE2 receptor regulation. Respir. Res..

[57] Wang Q., Khan N.A., Muthumalage T., Lawyer G.R., McDonough S.R., Chuang T.D., Gong M., Sundar I.K., Rehan V.K., Rahman I. (2019). Dysregulated repair and inflammatory responses by e-cigarette-derived inhaled nicotine and humectant propylene glycol in a sex-dependent manner in mouse lung. FASEB Bioadv..

[58] Naidu V., Zeki A.A., Sharma P. (2021). Sex differences in the induction of angiotensin converting enzyme 2 (ACE-2) in mouse lungs after e-cigarette vapor exposure and its relevance to COVID-19. J. Investig. Med..

[59] Lee A.C., Chakladar J., Li W.T., Chen C., Chang E.Y., Wang-Rodriguez J., Ongkeko W.M. (2020). Tobacco, but Not Nicotine and Flavor-Less Electronic Cigarettes, Induces ACE2 and Immune Dysregulation. Int. J. Mol. Sci..

[60] Sayed I.M., Masso-Silva J.A., Mittal A., Patel A., Lin E., Moshensky A., Shin J., Bojanowski C.M., Das S., Akuthota P. (2021). Inflammatory phenotype modulation in the respiratory tract and systemic circulation of e-cigarette users: A pilot study. Am. J. Physiol. Lung Cell. Mol. Physiol..

[61] Hickman E., Payton A., Duffney P., Wells H., Ceppe A.S., Brocke S., Bailey A., Rebuli M.E., Robinette C., Ring B. (2022). Biomarkers of Airway Immune Homeostasis Differ Significantly with Generation of E-Cigarettes. Am. J. Respir. Crit. Care Med..

[62] Gaschler G.J., Zavitz C.C., Bauer C.M., Skrtic M., Lindahl M., Robbins C.S., Chen B., Stampfli M.R. (2008). Cigarette smoke exposure attenuates cytokine production by mouse alveolar macrophages. Am. J. Respir. Cell Mol. Biol..

[63] Chen H., Cowan M.J., Hasday J.D., Vogel S.N., Medvedev A.E. (2007). Tobacco smoking inhibits expression of proinflammatory cytokines and activation of IL-1R-associated kinase, p38, and NF-kappaB in alveolar macrophages stimulated with TLR2 and TLR4 agonists. J. Immunol..

[64] Kent L., Smyth L., Clayton C., Scott L., Cook T., Stephens R., Fox S., Hext P., Farrow S., Singh D. (2008). Cigarette smoke extract induced cytokine and chemokine gene expression changes in COPD macrophages. Cytokine.

[65] Metcalfe H.J., Lea S., Hughes D., Khalaf R., Abbott-Banner K., Singh D. (2014). Effects of cigarette smoke on Toll-like receptor (TLR) activation of chronic obstructive pulmonary disease (COPD) macrophages. Clin. Exp. Immunol..

[66] Nordskog B.K., Fields W.R., Hellmann G.M. (2005). Kinetic analysis of cytokine response to cigarette smoke condensate by human endothelial and monocytic cells. Toxicology.

[67] Heulens N., Korf H., Mathyssen C., Everaerts S., De Smidt E., Dooms C., Yserbyt J., Gysemans C., Gayan-Ramirez G., Mathieu C. (2016). 1,25-Dihydroxyvitamin D Modulates Antibacterial and Inflammatory Response in Human Cigarette Smoke-Exposed Macrophages. PLoS ONE.

[68] Zhao J., Li X., Xie F., Yang Z., Pan X., Zhu M., Shang P., Nie C., Liu H., Xie J. (2017). Immunomodulatory effects of cigarette smoke condensate in mouse macrophage cell line. Int. J. Immunopathol. Pharmacol..

[69] Ma B., Kang M.J., Lee C.G., Chapoval S., Liu W., Chen Q., Coyle A.J., Lora J.M., Picarella D., Homer R.J. (2005). Role of CCR5 in IFN-gamma-induced and cigarette smoke-induced emphysema. J. Clin. Investig..

[70] Churg A., Wang R.D., Tai H., Wang X., Xie C., Dai J., Shapiro S.D., Wright J.L. (2003). Macrophage metalloelastase mediates acute cigarette smoke-induced inflammation via tumor necrosis factor-alpha release. Am. J. Respir. Crit. Care Med..

[71] Kuschner W.G., D’Alessandro A., Wong H., Blanc P.D. (1996). Dose-dependent cigarette smoking-related inflammatory responses in healthy adults. Eur. Respir. J..

[72] Bracke K.R., D‘Hulst A.I., Maes T., Demedts I.K., Moerloose K.B., Kuziel W.A., Joos G.F., Brusselle G.G. (2007). Cigarette smoke-induced pulmonary inflammation, but not airway remodelling, is attenuated in chemokine receptor 5-deficient mice. Clin. Exp. Allergy J. Br. Soc. Allergy Clin. Immunol..

[73] Jiang Y., Russell T.R., Graves D.T., Cheng H., Nong S.H., Levitz S.M. (1996). Monocyte chemoattractant protein 1 and interleukin-8 production in mononuclear cells stimulated by oral microorganisms. Infect. Immun..

[74] Perez M.F., Atuegwu N.C., Mortensen E.M., Oncken C. (2021). The inflammatory biomarker YKL-40 is elevated in the serum, but not the sputum, of E-cigarette users. Exp. Lung Res..

[75] Rankin G.D., Wingfors H., Uski O., Hedman L., Ekstrand-Hammarstrom B., Bosson J., Lundback M. (2019). The toxic potential of a fourth-generation E-cigarette on human lung cell lines and tissue explants. J. Appl. Toxicol..

[76] Antherieu S., Garat A., Beauval N., Soyez M., Allorge D., Garcon G., Lo-Guidice J.-M. (2017). Comparison of cellular and transcriptomic effects between electronic cigarette vapor and cigarette smoke in human bronchial epithelial cells. Toxicol. Vitr..

[77] Ishikawa S., Matsumura K., Kitamura N., Ishimori K., Takanami Y., Ito S. (2018). Application of a direct aerosol exposure system for the assessment of biological effects of cigarette smoke and novel tobacco product vapor on human bronchial epithelial cultures. Regul. Toxicol. Pharmacol..

[78] Bathrinarayanan P.V., Brown J.E., Marshall L.J., Leslie L.J. (2018). An investigation into E-cigarette cytotoxicity in-vitro using a novel 3D differentiated co-culture model of human airways. Toxicol. Vitr..

[79] Muthumalage T., Prinz M., Ansah K.O., Gerloff J., Sundar I.K., Rahman I. (2017). Inflammatory and Oxidative Responses Induced by Exposure to Commonly Used e-Cigarette Flavoring Chemicals and Flavored e-Liquids without Nicotine. Front. Physiol..

[80] Muthumalage T., Lamb T., Friedman M.R., Rahman I. (2019). E-cigarette flavored pods induce inflammation, epithelial barrier dysfunction, and DNA damage in lung epithelial cells and monocytes. Sci. Rep..

[81] Morris A.M., Leonard S.S., Fowles J.R., Boots T.E., Mnatsakanova A., Attfield K.R. (2021). Effects of E-Cigarette Flavoring Chemicals on Human Macrophages and Bronchial Epithelial Cells. Int. J. Environ. Res. Public Health.

[82] Dandrea T., Tu B., Blomberg A., Sandstrom T., Skold M., Eklund A., Cotgreave I. (1997). Differential inhibition of inflammatory cytokine release from cultured alveolar macrophages from smokers and non-smokers by NO_2_. Hum. Exp. Toxicol..

[83] Zhu X., Zhan Y., Gu Y., Huang Q., Wang T., Deng Z., Xie J. (2021). Cigarette Smoke Promotes Interleukin-8 Production in Alveolar Macrophages through the Reactive Oxygen Species/Stromal Interaction Molecule 1/Ca^2+^ Axis. Front. Physiol..

[84] da Silva C.O., Gicquel T., Daniel Y., Bartholo T., Vene E., Loyer P., Porto L.C., Lagente V., Victoni T. (2020). Alteration of immunophenotype of human macrophages and monocytes after exposure to cigarette smoke. Sci. Rep..

[85] Rumchev K., Brown H., Spickett J. (2007). Volatile organic compounds: Do they present a risk to our health?. Rev. Environ. Health.

[86] Sarigiannis D.A., Karakitsios S.P., Gotti A., Liakos I.L., Katsoyiannis A. (2011). Exposure to major volatile organic compounds and carbonyls in European indoor environments and associated health risk. Environ. Int..

[87] Yoon H.I., Hong Y.C., Cho S.H., Kim H., Kim Y.H., Sohn J.R., Kwon M., Park S.H., Cho M.H., Cheong H.K. (2010). Exposure to volatile organic compounds and loss of pulmonary function in the elderly. Eur. Respir. J..

[88] Alford K.L., Kumar N. (2021). Pulmonary Health Effects of Indoor Volatile Organic Compounds-A Meta-Analysis. Int. J. Environ. Res. Public Health.

[89] Wang F., Li C., Liu W., Jin Y. (2012). Effect of exposure to volatile organic compounds (VOCs) on airway inflammatory response in mice. J. Toxicol. Sci..

[90] Bonisch U., Bohme A., Kohajda T., Mogel I., Schutze N., von Bergen M., Simon J.C., Lehmann I., Polte T. (2012). Volatile organic compounds enhance allergic airway inflammation in an experimental mouse model. PLoS ONE.

[91] Wang F., Li C., Liu W., Jin Y., Guo L. (2014). Effects of subchronic exposure to low-dose volatile organic compounds on lung inflammation in mice. Environ. Toxicol..

[92] Fischader G., Roder-Stolinski C., Wichmann G., Nieber K., Lehmann I. (2008). Release of MCP-1 and IL-8 from lung epithelial cells exposed to volatile organic compounds. Toxicol. Vitr. An. Int. J. Publ. Assoc. BIBRA.

[93] Wang F., Li C., Liu W., Jin Y. (2014). Modulation of microRNA expression by volatile organic compounds in mouse lung. Environ. Toxicol..

[94] Lu F., Yu M., Chen C., Liu L., Zhao P., Shen B., Sun R. (2021). The Emission of VOCs and CO from Heated Tobacco Products, Electronic Cigarettes, and Conventional Cigarettes, and Their Health Risk. Toxics.

[95] Marco E., Grimalt J.O. (2015). A rapid method for the chromatographic analysis of volatile organic compounds in exhaled breath of tobacco cigarette and electronic cigarette smokers. J. Chromatogr. A.

[96] Papaefstathiou E., Stylianou M., Andreou C., Agapiou A. (2020). Breath analysis of smokers, non-smokers, and e-cigarette users. J. Chromatogr. B Anal. Technol. Biomed. Life Sci..

[97] Erythropel H.C., Davis L.M., de Winter T.M., Jordt S.E., Anastas P.T., O’Malley S.S., Krishnan-Sarin S., Zimmerman J.B. (2019). Flavorant-Solvent Reaction Products and Menthol in JUUL E-Cigarettes and Aerosol. Am. J. Prev. Med..

[98] Gschwend G., Jenkins C., Jones A., Kelso C., Morgan J. (2023). A Wide Range of Flavoring-Carrier Fluid Adducts Form in E-Cigarette Liquids. Chem. Res. Toxicol..

[99] Jabba S.V., Diaz A.N., Erythropel H.C., Zimmerman J.B., Jordt S.E. (2020). Chemical Adducts of Reactive Flavor Aldehydes Formed in E-Cigarette Liquids Are Cytotoxic and Inhibit Mitochondrial Function in Respiratory Epithelial Cells. Nicotine Tob. Res..

[100] Omaiye E.E., Luo W., McWhirter K.J., Pankow J.F., Talbot P. (2020). Electronic Cigarette Refill Fluids Sold Worldwide: Flavor Chemical Composition, Toxicity, and Hazard Analysis. Chem. Res. Toxicol..

[101] Kafferlein H.U., Broding H.C., Bunger J., Jettkant B., Koslitz S., Lehnert M., Marek E.M., Blaszkewicz M., Monse C., Weiss T. (2014). Human exposure to airborne aniline and formation of methemoglobin: A contribution to occupational exposure limits. Arch. Toxicol..

[102] Larsson F., Andersson P., Blomqvist P., Mellander B.E. (2017). Toxic fluoride gas emissions from lithium-ion battery fires. Sci. Rep..

[103] Ma J., Xia Q., Fu P.P., Lin G. (2018). Pyrrole-protein adducts—A biomarker of pyrrolizidine alkaloid-induced hepatotoxicity. J. Food Drug Anal..

[104] Rezaei A., Rezaei M.R., Sayadi M.H. (2021). Enhanced 3,5-dimethylphenol photodegradation via adsorption-photocatalysis synergy using FSTRG nanohybrid catalyst. J. Mol. Liq..

[105] Gupta G.D., Misra A., Agarwal D.K. (1991). Inhalation toxicity of furfural vapours: An assessment of biochemical response in rat lungs. J. Appl. Toxicol..

[106] Cirillo S., Urena J.F., Lambert J.D., Vivarelli F., Canistro D., Paolini M., Cardenia V., Rodriguez-Estrada M.T., Richie J.P., Elias R.J. (2019). Impact of electronic cigarette heating coil resistance on the production of reactive carbonyls, reactive oxygen species and induction of cytotoxicity in human lung cancer cells in vitro. Regul. Toxicol. Pharmacol..

[107] St. Helen G., Liakoni E., Nardone N., Addo N., Jacob P., Benowitz N.L. (2020). Comparison of Systemic Exposure to Toxic and/or Carcinogenic Volatile Organic Compounds (VOC) during Vaping, Smoking, and Abstention. Cancer Prev. Res..

[108] Cunningham A., McAdam K., Thissen J., Digard H. (2020). The Evolving E-cigarette: Comparative Chemical Analyses of E-cigarette Vapor and Cigarette Smoke. Front. Toxicol..

[109] Ween M.P., White J.B., Tran H.B., Mukaro V., Jones C., Macowan M., Hodge G., Trim P.J., Snel M.F., Hodge S.J. (2021). The role of oxidised self-lipids and alveolar macrophage CD1b expression in COPD. Sci. Rep..

