# Supplementary material for: Dual Exposure to E-Cigarette Vapour and Cigarette Smoke Results in Poorer Airway Cell, Monocyte, and Macrophage Function Than Single Exposure
Source: Int J Mol Sci. 2024 May 31;25(11):6071. doi: 10.3390/ijms25116071 (PMC11173218; doi:10.3390/ijms25116071)
Supplement: Supplementary file 1 [file ijms-25-06071-s001.zip › ijms-2972627-supplementary.pdf]

Table S1. Mass spectrometry analysis of VOCs in single and dual extract samples frozen immediately after collection

**Chemical**	**CSE**	**PGVG**	**CSE IN PGVG**	**PGVG in CSE**
2,2-dimethyl-propanal	DETECTED		NO LONGER DETECTED	NO LONGER DETECTED
3-propoxy-1-propene				
2-Pentanone	DETECTED		NO LONGER DETECTED	NO LONGER DETECTED
Allyl pentyl ester oxalic acid	DETECTED		NO LONGER DETECTED	NO LONGER DETECTED
Acetic acid ethenyl ester	DETECTED		NO LONGER DETECTED	NO LONGER DETECTED
Pyridine	DETECTED		STILL DETECTED	STILL DETECTED
Pyrrole	DETECTED		STILL DETECTED	STILL DETECTED
Cyclopentanone	DETECTED		STILL DETECTED	STILL DETECTED
Isopropyl Alcohol				
Propylene Glycol		DETECTED	STILL DETECTED	STILL DETECTED
Methylpyrazine	DETECTED		NO LONGER DETECTED	NO LONGER DETECTED
2-Cyclopenten-1-one				NEW
Furfural				
2-oxo-3-cyclopentene-1-acetaldehyde	DETECTED		NO LONGER DETECTED	NO LONGER DETECTED
3-methylpyridine	DETECTED		NO LONGER DETECTED	NO LONGER DETECTED
Aniline				
Diacetate 1,1-ethanediol			NEW	
2,5-dimethylpyrazine/2,6-dimethylpyrazine/2,5-Dimethylpyrimidine	DETECTED		STILL DETECTED	STILL DETECTED
2-methyl-2-cyclopenten-1-one	DETECTED		STILL DETECTED	STILL DETECTED
2-ethenyl-pyridine	DETECTED		NO LONGER DETECTED	NO LONGER DETECTED
Trimethylpyrazine				
3-methyl-2-cyclopenten-1-one	DETECTED		NO LONGER DETECTED	NO LONGER DETECTED
2,3-dihydroxy-propanal				
Methyl Alcohol				
Methylglyoxal				
1,3-dihydroxy-2-Propanone	DETECTED	DETECTED	STILL DETECTED	STILL DETECTED
1-(3H-Imidazol-4-yl)-ethanone	DETECTED		NO LONGER DETECTED	STILL DETECTED
3,4-dimethyl-2-cyclopenten-1-one/2,3-dimethyl-2-cyclopenten-1-one	DETECTED		STILL DETECTED	STILL DETECTED
Phenol/Vinyl furan	DETECTED		STILL DETECTED	STILL DETECTED

2,5,6,7-tetrahydro-3H-Cyclopenta[c]pyridazin-3-one/3-methylene-2-oxo-cyclohexanecarboxylic acid, methyl ester				
1-ethoxy-2-methylbenzene				
Glycerin	DETECTED	DETECTED	STILL DETECTED	STILL DETECTED
2-Acetyl-5-methylfuran/2-methoxyphenol/Mequinol	DETECTED		STILL DETECTED	STILL DETECTED
2-methylphenol	DETECTED		STILL DETECTED	STILL DETECTED
4-methylphenol	DETECTED		STILL DETECTED	STILL DETECTED
N-methyl-1-Octanamine				
Acetaldehyde				
3,5-dimethylphenol				NEW
1-methoxy-4-methyl-benzene	DETECTED		NO LONGER DETECTED	NO LONGER DETECTED
3-pyridinol	DETECTED		STILL DETECTED	STILL DETECTED
3-pyridinamine				
4-ethylphenol	DETECTED		NO LONGER DETECTED	REDUCED DETECTION
Phosphoryl fluoride				
3-(1-methyl-2-pyrrolidinyl)pyridine	DETECTED	DETECTED	STILL DETECTED	STILL DETECTED
Indole/m-Aminophenylacetylene	DETECTED		STILL DETECTED	STILL DETECTED
3-(3,4-dihydro-2H-pyrrol-5-yl)-pyridine	DETECTED		STILL DETECTED	STILL DETECTED
Vanillin/2-hydroxy-4-methoxy-benzaldehyde/3-hydroxy-4-methoxy-benzaldehyde				STILL DETECTED
N-methyl-benzeneacetamide				
Benzeneacetamide			NEW	
3-methyl-1-phenyl-1H-pyrazole				
2,2'-Bipyrazine				
2,3'-Dipyridyl	DETECTED		NO LONGER DETECTED	STILL DETECTED

STILL DETECTED = detected in single extract and also in dual extract. NO LONGER DETECTED = detected in single extract and no longer detected in dual extract. REDUCED DETECTION = full detection in single extract, only trace detection in dual extract. NEW = only detected in dual extract and not detected in single extracts. Changes in dual extracts highlighted in grey.

Table S2. Mass spectrometry analysis of VOCs in in single and dual extract samples incubated at 37°C 5% CO2 for 24 hrs immediately after collection

**Chemical**	**CSE**	**PGVG**	**CSE IN PGVG**	**PGVG in CSE**
2,2-dimethyl-propanal				
3-propoxy-1-propene				
2-Pentanone				
Allyl pentyl ester oxalic acid				
Acetic acid ethenyl ester				
Pyridine	DETECTED		STILL DETECTED	STILL DETECTED
Pyrrole			NEW	NEW
Cyclopentanone				NEW
Isopropyl Alcohol				
Propylene Glycol		DETECTED	STILL DETECTED	STILL DETECTED
Methylpyrazine	DETECTED		NO LONGER DETECTED	NO LONGER DETECTED
2-Cyclopenten-1-one				
Furfural			NEW	NEW
2-oxo-3-cyclopentene-1-acetaldehyde				
3-methylpyridine				
Aniline				
Diacetate 1,1-ethanediol				
2,5-dimethylpyrazine/2,6-dimethylpyrazine/2,5-Dimethylpyrimidine	DETECTED		STILL DETECTED	STILL DETECTED
2-methyl-2-cyclopenten-1-one	DETECTED		STILL DETECTED	STILL DETECTED
2-ethenyl-pyridine				
Trimethylpyrazine		DETECTED	STILL DETECTED	STILL DETECTED
3-methyl-2-cyclopenten-1-one				
2,3-dihydroxy-propanal				
Methyl Alcohol	DETECTED		NO LONGER DETECTED	NO LONGER DETECTED
Methylglyoxal				
1,3-dihydroxy-2-Propanone	DETECTED	DETECTED	STILL DETECTED	STILL DETECTED
1-(3H-Imidazol-4-yl)-ethanone				
3,4-dimethyl-2-cyclopenten-1-one/2,3-dimethyl-2-cyclopenten-1-one	DETECTED		NO LONGER DETECTED	STILL DETECTED
Phenol/Vinyl furan	DETECTED		STILL DETECTED	STILL DETECTED

2,5,6,7-tetrahydro-3H-Cyclopenta[c]pyridazin-3-one/3-methylene-2-oxo-cyclohexanecarboxylic acid, methyl ester		DETECTED	STILL DETECTED	STILL DETECTED
1-ethoxy-2-methylbenzene				
Glycerin	DETECTED	DETECTED	STILL DETECTED	STILL DETECTED
2-Acetyl-5-methylfuran/2-methoxyphenol/Mequinol		DETECTED	STILL DETECTED	STILL DETECTED
2-methylphenol	DETECTED		STILL DETECTED	STILL DETECTED
4-methylphenol	DETECTED		STILL DETECTED	STILL DETECTED
N-methyl-1-Octanamine				
Acetaldehyde				
3,5-dimethylphenol				
1-methoxy-4-methyl-benzene				
3-pyridinol	DETECTED		NO LONGER DETECTED	STILL DETECTED
3-pyridinamine				
4-ethylphenol				
Phosphoryl fluoride				
3-(1-methyl-2-pyrrolidinyl)pyridine	DETECTED	DETECTED	STILL DETECTED	STILL DETECTED
Indole/m-Aminophenylacetylene	DETECTED		STILL DETECTED	STILL DETECTED
3-(3,4-dihydro-2H-pyrrol-5-yl)-pyridine	DETECTED		STILL DETECTED	STILL DETECTED
Vanillin/2-hydroxy-4-methoxy-benzaldehyde/3-hydroxy-4-methoxy-benzaldehyde		DETECTED	STILL DETECTED	STILL DETECTED
N-methyl-benzeneacetamide				
Benzeneacetamide		DETECTED	STILL DETECTED	STILL DETECTED
3-methyl-1-phenyl-1H-pyrazole				STILL DETECTED
2,2'-Bipyrazine				
2,3'-Dipyridyl	DETECTED		STILL DETECTED	STILL DETECTED

STILL DETECTED = detected in 0hr extract and also in 4hr2 extract. NO LONGER DETECTED = detected in 0hr extract and no longer detected in 24hr extract. NEW = only detected in 24hr extract and not detected in 0hr extracts. Changes in 24hr extracts highlighted in grey.

Table S3. Mass spectrometry analysis of VOCs in CSE samples frozen immediately after collection and those incubated at 37°C 5% CO_2 for 24 hrs immediately after collection

**Chemical**	**CSE 0HR**	**CSE 24HR**
2,2-dimethyl-propanal	DETECTED	NO LONGER DETECTED
3-propoxy-1-propene		
2-Pentanone	DETECTED	NO LONGER DETECTED
Allyl pentyl ester oxalic acid	DETECTED	NO LONGER DETECTED
Acetic acid ethenyl ester	DETECTED	NO LONGER DETECTED
Pyridine	DETECTED	STILL DETECTED
Pyrrole	DETECTED	NO LONGER DETECTED
Cyclopentanone	DETECTED	NO LONGER DETECTED
Isopropyl Alcohol		
Propylene Glycol		
Methylpyrazine	DETECTED	STILL DETECTED
2-Cyclopenten-1-one		
Furfural		
2-oxo-3-cyclopentene-1-acetaldehyde	DETECTED	NO LONGER DETECTED
3-methylpyridine	DETECTED	NO LONGER DETECTED
Aniline		
Diacetate 1,1-ethanediol		
2,5-dimethylpyrazine/2,6-dimethylpyrazine/2,5-Dimethylpyrimidine	DETECTED	STILL DETECTED
2-methyl-2-cyclopenten-1-one	DETECTED	STILL DETECTED
2-ethenyl-pyridine	DETECTED	NO LONGER DETECTED
Trimethylpyrazine		
3-methyl-2-cyclopenten-1-one	DETECTED	NO LONGER DETECTED
2,3-dihydroxy-propanal		
Methyl Alcohol		NEW
Methylglyoxal		
1,3-dihydroxy-2-Propanone	DETECTED	STILL DETECTED
1-(3H-Imidazol-4-yl)-ethanone	DETECTED	NO LONGER DETECTED
3,4-dimethyl-2-cyclopenten-1-one/2,3-dimethyl-2-cyclopenten-1-one	DETECTED	STILL DETECTED
Phenol/Vinyl furan	DETECTED	STILL DETECTED
2,5,6,7-tetrahydro-3H-Cyclopenta[c]pyridazin-3-one/3-methylene-2-oxo-cyclohexanecarboxylic acid, methyl ester		
1-ethoxy-2-methylbenzene		
Glycerin	DETECTED	STILL DETECTED
2-Acetyl-5-methylfuran/2-methoxyphenol/Mequinol	DETECTED	
2-methylphenol	DETECTED	STILL DETECTED
4-methylphenol	DETECTED	STILL DETECTED
N-methyl-1-Octanamine		
Acetaldehyde		
3,5-dimethylphenol		
1-methoxy-4-methyl-benzene	DETECTED	NO LONGER DETECTED
3-pyridinol	DETECTED	STILL DETECTED
3-pyridinamine		
4-ethylphenol	DETECTED	NO LONGER DETECTED

Phosphoryl fluoride		
3-(1-methyl-2-pyrrolidinyl)pyridine	DETECTED	STILL DETECTED
Indole/m-Aminophenylacetylene	DETECTED	STILL DETECTED
3-(3,4-dihydro-2H-pyrrol-5-yl)-pyridine	DETECTED	STILL DETECTED
Vanillin/2-hydroxy-4-methoxy-benzaldehyde/3-hydroxy-4-methoxy-benzaldehyde		
N-methyl-benzeneacetamide		
Benzeneacetamide		
3-methyl-1-phenyl-1H-pyrazole		
2,2'-Bipyrazine		
2,3'-Dipyridyl	DETECTED	STILL DETECTED

STILL DETECTED = detected in 0hr extract and also in 4hr2 extract. NO LONGER DETECTED = detected in 0hr extract and no longer detected in 24hr extract. NEW = only detected in 24hr extract and not detected in 0hr extracts. Changes in 24hr extracts highlighted in grey.

Table S4. Mass spectrometry analysis of VOCs in Chocolate EVE samples frozen immediately after collection and those incubated at 37°C 5% CO_2 for 24 hrs immediately after collection

**Chemical**	**CHOC 0HR**	**CHOC 24HR**
2,2-dimethyl-propanal		
3-propoxy-1-propene		
2-Pentanone		
Allyl pentyl ester oxalic acid		
Acetic acid ethenyl ester		
Pyridine		
Pyrrole		
Cyclopentanone		
Isopropyl Alcohol		
Propylene Glycol	DETECTED	DETECTED
Methylpyrazine		
2-Cyclopenten-1-one		
Furfural		
2-oxo-3-cyclopentene-1-acetaldehyde		
3-methylpyridine		
Aniline		
Diacetate 1,1-ethanediol		
2,5-dimethylpyrazine/2,6-dimethylpyrazine/2,5-Dimethylpyrimidine	DETECTED	NO LONGER DETECTED
2-methyl-2-cyclopenten-1-one		
2-ethenyl-pyridine		
Trimethylpyrazine	DETECTED	NO LONGER DETECTED
3-methyl-2-cyclopenten-1-one		
2,3-dihydroxy-propanal		
Methyl Alcohol		
Methylglyoxal		NEW
1,3-dihydroxy-2-Propanone	DETECTED	NO LONGER DETECTED
1-(3H-Imidazol-4-yl)-ethanone		
3,4-dimethyl-2-cyclopenten-1-one/2,3-dimethyl-2-cyclopenten-1-one		
Phenol/Vinyl furan		
2,5,6,7-tetrahydro-3H-Cyclopenta[c]pyridazin-3-one/3-methylene-2-oxo-cyclohexanecarboxylic acid, methyl ester	DETECTED	NO LONGER DETECTED
1-ethoxy-2-methylbenzene		
Glycerin	DETECTED	DETECTED
2-Acetyl-5-methylfuran/2-methoxyphenol/Mequinol	DETECTED	NO LONGER DETECTED
2-methylphenol		
4-methylphenol		
N-methyl-1-Octanamine		
Acetaldehyde		
3,5-dimethylphenol		
1-methoxy-4-methyl-benzene		
3-pyridinol		
3-pyridinamine		
4-ethylphenol		
Phosphoryl fluoride		

3-(1-methyl-2-pyrrolidinyl)pyridine		NEW
Indole/m-Aminophenylacetylene		
3-(3,4-dihydro-2H-pyrrol-5-yl)-pyridine		
Vanillin/2-hydroxy-4-methoxy-benzaldehyde/3-hydroxy-4-methoxy-benzaldehyde	DETECTED	NO LONGER DETECTED
N-methyl-benzeneacetamide		
Benzeneacetamide		
3-methyl-1-phenyl-1H-pyrazole		
2,2'-Bipyrazine		
2,3'-Dipyridyl		

STILL DETECTED = detected in 0hr extract and also in 4hr2 extract. NO LONGER DETECTED = detected in 0hr extract and no longer detected in 24hr extract. NEW = only detected in 24hr extract and not detected in 0hr extracts. Changes in 24hr extracts highlighted in grey.

Table S5. Mass spectrometry analysis of VOCs in CSE made into Chocolate EVE samples frozen immediately after collection and those incubated at 37°C 5% CO_2 for 24 hrs immediately after collection

**Chemical**	**CSE in CHOC 0HR**	**CSE in CHOC 24HR**
2,2-dimethyl-propanal	TRACE	NO LONGER DETECTED
3-propoxy-1-propene		
2-Pentanone		
Allyl pentyl ester oxalic acid	TRACE	NO LONGER DETECTED
Acetic acid ethenyl ester		
Pyridine	DETECTED	DETECTED
Pyrrole	DETECTED	DETECTED
Cyclopentanone	DETECTED	DETECTED
Isopropyl Alcohol		
Propylene Glycol	DETECTED	DETECTED
Methylpyrazine		
2-Cyclopenten-1-one		
Furfural		
2-oxo-3-cyclopentene-1-acetaldehyde		
3-methylpyridine		
Aniline	DETECTED	NO LONGER DETECTED
Diacetate 1,1-ethanediol		
2,5-dimethylpyrazine/2,6-dimethylpyrazine/2,5-Dimethylpyrimidine	DETECTED	DETECTED
2-methyl-2-cyclopenten-1-one	DETECTED	DETECTED
2-ethenyl-pyridine		
Trimethylpyrazine	DETECTED	NO LONGER DETECTED
3-methyl-2-cyclopenten-1-one		NEW
2,3-dihydroxy-propanal		
Methyl Alcohol		
Methylglyoxal		
1,3-dihydroxy-2-Propanone	DETECTED	DETECTED
1-(3H-Imidazol-4-yl)-ethanone		
3,4-dimethyl-2-cyclopenten-1-one/2,3-dimethyl-2-cyclopenten-1-one	DETECTED	DETECTED
Phenol/Vinyl furan	DETECTED	DETECTED
2,5,6,7-tetrahydro-3H-Cyclopenta[c]pyridazin-3-one/3-methylene-2-oxo-cyclohexanecarboxylic acid, methyl ester	DETECTED	NO LONGER DETECTED
1-ethoxy-2-methylbenzene		
Glycerin	DETECTED	DETECTED
2-Acetyl-5-methylfuran/2-methoxyphenol/Mequinol	DETECTED	DETECTED
2-methylphenol	DETECTED	DETECTED
4-methylphenol	DETECTED	DETECTED
N-methyl-1-Octanamine		
Acetaldehyde		
3,5-dimethylphenol		
1-methoxy-4-methyl-benzene		
3-pyridinol	DETECTED	NO LONGER DETECTED
3-pyridinamine		
4-ethylphenol		

Phosphoryl fluoride		
3-(1-methyl-2-pyrrolidinyl)pyridine	DETECTED	DETECTED
Indole/m-Aminophenylacetylene	DETECTED	DETECTED
3-(3,4-dihydro-2H-pyrrol-5-yl)-pyridine	DETECTED	DETECTED
Vanillin/2-hydroxy-4-methoxy-benzaldehyde/3-hydroxy-4-methoxy-benzaldehyde	DETECTED	DETECTED
N-methyl-benzeneacetamide		
Benzeneacetamide		NEW
3-methyl-1-phenyl-1H-pyrazole		
2,2'-Bipyrazine	DETECTED	NO LONGER DETECTED
2,3'-Dipyridyl	DETECTED	DETECTED

STILL DETECTED = detected in 0hr extract and also in 4hr2 extract. NO LONGER DETECTED = detected in 0hr extract and no longer detected in 24hr extract. NEW = only detected in 24hr extract and not detected in 0hr extracts. Changes in 24hr extracts highlighted in grey.

Table S6. Mass spectrometry analysis of VOCs in Chocolate EVE made into CSE samples frozen immediately after collection and those incubated at 37°C 5% CO_2 for 24 hrs immediately after collection

Chemical	CHOC IN CSE 0HR	CHOC IN CSE 24HR
2,2-dimethyl-propanal		
3-propoxy-1-propene		
2-Pentanone		
Allyl pentyl ester oxalic acid		
Acetic acid ethenyl ester		
Pyridine	DETECTED	STILL DETECTED
Pyrrole	DETECTED	STILL DETECTED
Cyclopentanone		
Isopropyl Alcohol		
Propylene Glycol	DETECTED	STILL DETECTED
Methylpyrazine		
2-Cyclopenten-1-one		
Furfural		
2-oxo-3-cyclopentene-1-acetaldehyde	DETECTED	NO LONGER DETECTED
3-methylpyridine	DETECTED	NO LONGER DETECTED
Aniline		
Diacetate 1,1-ethanediol		
2,5-dimethylpyrazine/2,6-dimethylpyrazine/2,5-Dimethylpyrimidine	DETECTED	STILL DETECTED
2-methyl-2-cyclopenten-1-one	DETECTED	STILL DETECTED
2-ethenyl-pyridine		
Trimethylpyrazine	DETECTED	NO LONGER DETECTED
3-methyl-2-cyclopenten-1-one		NEW
2,3-dihydroxy-propanal		
Methyl Alcohol		
Methylglyoxal		
1,3-dihydroxy-2-Propanone	DETECTED	STILL DETECTED
1-(3H-Imidazol-4-yl)-ethanone	DETECTED	NO LONGER DETECTED
3,4-dimethyl-2-cyclopenten-1-one/2,3-dimethyl-2-cyclopenten-1-one		
Phenol/Vinyl furan	DETECTED	STILL DETECTED
2,5,6,7-tetrahydro-3H-Cyclopenta[c]pyridazin-3-one/3-methylene-2-oxo-cyclohexanecarboxylic acid, methyl ester		
1-ethoxy-2-methylbenzene	DETECTED	NO LONGER DETECTED
Glycerin	DETECTED	STILL DETECTED
2-Acetyl-5-methylfuran/2-methoxyphenol/Mequinol	DETECTED	STILL DETECTED
2-methylphenol	DETECTED	STILL DETECTED
4-methylphenol	DETECTED	STILL DETECTED
N-methyl-1-Octanamine		
Acetaldehyde		
3,5-dimethylphenol		
1-methoxy-4-methyl-benzene		
3-pyridinol	DETECTED	STILL DETECTED
3-pyridinamine	DETECTED	NO LONGER DETECTED

4-ethylphenol		
Phosphoryl fluoride	DETECTED	NO LONGER DETECTED
3-(1-methyl-2-pyrrolidinyl)pyridine	DETECTED	STILL DETECTED
Indole/m-Aminophenylacetylene	DETECTED	STILL DETECTED
3-(3,4-dihydro-2H-pyrrol-5-yl)-pyridine	DETECTED	STILL DETECTED
Vanillin/2-hydroxy-4-methoxy-benzaldehyde/3-hydroxy-4-methoxy-benzaldehyde	DETECTED	NO LONGER DETECTED
N-methyl-benzeneacetamide	DETECTED	NO LONGER DETECTED
Benzeneacetamide		NEW
3-methyl-1-phenyl-1H-pyrazole		
2,2'-Bipyrazine		
2,3'-Dipyridyl	DETECTED	STILL DETECTED

STILL DETECTED = detected in 0hr extract and also in 4hr2 extract. NO LONGER DETECTED = detected in 0hr extract and no longer detected in 24hr extract. NEW = only detected in 24hr extract and not detected in 0hr extracts. Changes in 24hr extracts highlighted in grey.

Table S7. Mass spectrometry analysis of VOCs in PGVE EVE samples frozen immediately after collection and those incubated at 37°C 5% CO_2 for 24 hrs immediately after collection

**Chemical**	**PGVG 0HR**	**PGVG 24HR**
2,2-dimethyl-propanal		
3-propoxy-1-propene		
2-Pentanone		
Allyl pentyl ester oxalic acid		
Acetic acid ethenyl ester		
Pyridine		
Pyrrole		
Cyclopentanone		
Isopropyl Alcohol		
Propylene Glycol	DETECTED	STILL DETECTED
Methylpyrazine		
2-Cyclopenten-1-one		
Furfural		
2-oxo-3-cyclopentene-1-acetaldehyde		
3-methylpyridine		
Aniline		
Diacetate 1,1-ethanediol		
2,5-dimethylpyrazine/2,6-dimethylpyrazine/2,5-Dimethylpyrimidine		
2-methyl-2-cyclopenten-1-one		
2-ethenyl-pyridine		
Trimethylpyrazine		NEW
3-methyl-2-cyclopenten-1-one		
2,3-dihydroxy-propanal		
Methyl Alcohol		
Methylglyoxal		
1,3-dihydroxy-2-Propanone	DETECTED	STILL DETECTED
1-(3H-Imidazol-4-yl)-ethanone		
3,4-dimethyl-2-cyclopenten-1-one/2,3-dimethyl-2-cyclopenten-1-one		
Phenol/Vinyl furan		
2,5,6,7-tetrahydro-3H-Cyclopenta[c]pyridazin-3-one/3-methylene-2-oxo-cyclohexanecarboxylic acid, methyl ester		NEW
1-ethoxy-2-methylbenzene		
Glycerin	DETECTED	STILL DETECTED
2-Acetyl-5-methylfuran/2-methoxyphenol/Mequinol		NEW
2-methylphenol		
4-methylphenol		
N-methyl-1-Octanamine		
Acetaldehyde		
3,5-dimethylphenol		
1-methoxy-4-methyl-benzene		
3-pyridinol		
3-pyridinamine		
4-ethylphenol		
Phosphoryl fluoride		

3-(1-methyl-2-pyrrolidinyl)pyridine	DETECTED	STILL DETECTED
Indole/m-Aminophenylacetylene		
3-(3,4-dihydro-2H-pyrrol-5-yl)-pyridine		
Vanillin/2-hydroxy-4-methoxy-benzaldehyde/3-hydroxy-4-methoxy-benzaldehyde		NEW
N-methyl-benzeneacetamide		
Benzeneacetamide		NEW
3-methyl-1-phenyl-1H-pyrazole		
2,2'-Bipyrazine		
2,3'-Dipyridyl		

STILL DETECTED = detected in 0hr extract and also in 4hr2 extract. NO LONGER DETECTED = detected in 0hr extract and no longer detected in 24hr extract. NEW = only detected in 24hr extract and not detected in 0hr extracts. Changes in 24hr extracts highlighted in grey.

Table S8. Mass spectrometry analysis of VOCs in CSE made into PGVG EVE samples frozen immediately after collection and those incubated at 37°C 5% CO_2 for 24 hrs immediately after collection

**Chemical**	**CSE IN PGVG 0HR**	**CSE IN PGVG 24HR**
2,2-dimethyl-propanal		
3-propoxy-1-propene		
2-Pentanone		
Allyl pentyl ester oxalic acid		
Acetic acid ethenyl ester		
Pyridine	DETECTED	STILL DETECTED
Pyrrole	DETECTED	STILL DETECTED
Cyclopentanone	DETECTED	
Isopropyl Alcohol		
Propylene Glycol	DETECTED	STILL DETECTED
Methylpyrazine		
2-Cyclopenten-1-one		
Furfural		NEW
2-oxo-3-cyclopentene-1-acetaldehyde		
3-methylpyridine		
Aniline		
Diacetate 1,1-ethanediol	DETECTED	NO LONGER DETECTED
2,5-dimethylpyrazine/2,6-dimethylpyrazine/2,5-Dimethylpyrimidine	DETECTED	STILL DETECTED
2-methyl-2-cyclopenten-1-one	DETECTED	STILL DETECTED
2-ethenyl-pyridine		
Trimethylpyrazine		NEW
3-methyl-2-cyclopenten-1-one		
2,3-dihydroxy-propanal		
Methyl Alcohol		
Methylglyoxal		
1,3-dihydroxy-2-Propanone	DETECTED	STILL DETECTED
1-(3H-Imidazol-4-yl)-ethanone		
3,4-dimethyl-2-cyclopenten-1-one/2,3-dimethyl-2-cyclopenten-1-one	DETECTED	NO LONGER DETECTED
Phenol/Vinyl furan	DETECTED	STILL DETECTED
2,5,6,7-tetrahydro-3H-Cyclopenta[c]pyridazin-3-one/3-methylene-2-oxo-cyclohexanecarboxylic acid, methyl ester		NEW
1-ethoxy-2-methylbenzene		
Glycerin	DETECTED	STILL DETECTED
2-Acetyl-5-methylfuran/2-methoxyphenol/Mequinol	DETECTED	STILL DETECTED
2-methylphenol	DETECTED	STILL DETECTED
4-methylphenol	DETECTED	STILL DETECTED
N-methyl-1-Octanamine		
Acetaldehyde		
3,5-dimethylphenol		
1-methoxy-4-methyl-benzene		
3-pyridinol	DETECTED	NO LONGER DETECTED
3-pyridinamine		
4-ethylphenol		

Phosphoryl fluoride		
3-(1-methyl-2-pyrrolidinyl)pyridine	DETECTED	STILL DETECTED
Indole/m-Aminophenylacetylene	DETECTED	STILL DETECTED
3-(3,4-dihydro-2H-pyrrol-5-yl)-pyridine	DETECTED	STILL DETECTED
Vanillin/2-hydroxy-4-methoxy-benzaldehyde/3-hydroxy-4-methoxy-benzaldehyde		NEW
N-methyl-benzeneacetamide		
Benzeneacetamide	DETECTED	STILL DETECTED
3-methyl-1-phenyl-1H-pyrazole		
2,2'-Bipyrazine		
2,3'-Dipyridyl		NEW

STILL DETECTED = detected in 0hr extract and also in 4hr2 extract. NO LONGER DETECTED = detected in 0hr extract and no longer detected in 24hr extract. NEW = only detected in 24hr extract and not detected in 0hr extracts. Changes in 24hr extracts highlighted in grey.

Table S9. Mass spectrometry analysis of VOCs in PGVE EVE made into CSE samples frozen immediately after collection and those incubated at 37°C 5% CO_2 for 24 hrs immediately after collection

Chemical	PGVG in CSE 0HR	PGVG in CSE 24HR
2,2-dimethyl-propanal		
3-propoxy-1-propene		
2-Pentanone		
Allyl pentyl ester oxalic acid		
Acetic acid ethenyl ester		
Pyridine	DETECTED	STILL DETECTED
Pyrrole	DETECTED	STILL DETECTED
Cyclopentanone	DETECTED	STILL DETECTED
Isopropyl Alcohol		
Propylene Glycol	DETECTED	STILL DETECTED
Methylpyrazine		
2-Cyclopenten-1-one	DETECTED	NO LONGER DETECTED
Furfural		NEW
2-oxo-3-cyclopentene-1-acetaldehyde		
3-methylpyridine		
Aniline		
Diacetate 1,1-ethanediol		
2,5-dimethylpyrazine/2,6-dimethylpyrazine/2,5-Dimethylpyrimidine	DETECTED	STILL DETECTED
2-methyl-2-cyclopenten-1-one	DETECTED	STILL DETECTED
2-ethenyl-pyridine		
Trimethylpyrazine		NEW
3-methyl-2-cyclopenten-1-one		
2,3-dihydroxy-propanal		
Methyl Alcohol		
Methylglyoxal		
1,3-dihydroxy-2-Propanone	DETECTED	STILL DETECTED
1-(3H-Imidazol-4-yl)-ethanone	DETECTED	NO LONGER DETECTED
3,4-dimethyl-2-cyclopenten-1-one/2,3-dimethyl-2-cyclopenten-1-one	DETECTED	STILL DETECTED
Phenol/Vinyl furan	DETECTED	STILL DETECTED
2,5,6,7-tetrahydro-3H-Cyclopenta[c]pyridazin-3-one/3-methylene-2-oxo-cyclohexanecarboxylic acid, methyl ester		NEW
1-ethoxy-2-methylbenzene		
Glycerin	DETECTED	STILL DETECTED
2-Acetyl-5-methylfuran/2-methoxyphenol/Mequinol	DETECTED	STILL DETECTED
2-methylphenol	DETECTED	STILL DETECTED
4-methylphenol	DETECTED	STILL DETECTED
N-methyl-1-Octanamine		
Acetaldehyde		
3,5-dimethylphenol	DETECTED	NO LONGER DETECTED
1-methoxy-4-methyl-benzene		
3-pyridinol	DETECTED	STILL DETECTED
3-pyridinamine		

4-ethylphenol		
Phosphoryl fluoride		
3-(1-methyl-2-pyrrolidinyl)pyridine	DETECTED	STILL DETECTED
Indole/m-Aminophenylacetylene	DETECTED	STILL DETECTED
3-(3,4-dihydro-2H-pyrrol-5-yl)-pyridine	DETECTED	STILL DETECTED
Vanillin/2-hydroxy-4-methoxy-benzaldehyde/3-hydroxy-4-methoxy-benzaldehyde	DETECTED	STILL DETECTED
N-methyl-benzeneacetamide		
Benzeneacetamide		NEW
3-methyl-1-phenyl-1H-pyrazole		NEW
2,2'-Bipyrazine		
2,3'-Dipyridyl	DETECTED	STILL DETECTED

STILL DETECTED = detected in 0hr extract and also in 4hr2 extract. NO LONGER DETECTED = detected in 0hr extract and no longer detected in 24hr extract. NEW = only detected in 24hr extract and not detected in 0hr extracts. Changes in 24hr extracts highlighted in grey.
